# Obesity, Metabolic Modulation, and Arrhythmias: From Adipose Tissue Dysfunction to a Modifiable Arrhythmogenic Substrate

**DOI:** 10.3390/biomedicines14102269

**Published:** 2026-10-07

**Authors:** Margherita Tiezzi, Federica Valli, Andrea Mengozzi, Letizia Ferroni, Sofia Brasini, Giorgia Capirossi, Paolo Gentileschi, Elena Tremoli

**Affiliations:** 1 GVM Care & Research, IRCCS Maria Cecilia Hospital, 48033 Cotignola, Italy; 2 Dipartimento di Scienze Chirurgiche, Università di Roma Tor Vergata, 00133 Rome, Italy

**Keywords:** obesity, arrhythmias, epicardial adipose tissue, visceral adipose tissue, extracellular vesicles, miRNAs, weight reduction

## Abstract

The obese patient with arrhythmia represents a major challenge in cardiology. In these patients, multiple mechanisms converge to create a pro-arrhythmic milieu that remains poorly understood and only partially controllable. The anatomical and functional remodeling of adipose tissue, local inflammation, metabolic alterations, and the frequent presence of associated comorbidities lead to structural, functional, and electrical cardiac changes, resulting in a pro-arrhythmic substrate. The coexistence of these multiple contributing factors may reduce the efficacy of currently available antiarrhythmic therapies in this specific population. Although these obesity-related alterations are not currently established therapeutic targets, they may represent promising targets for future interventions to improve patients’ outcomes. In this narrative review, we summarize the main scientific evidence regarding the pro-arrhythmic cardiac alterations associated with obesity, focusing separately on structural and electrical remodeling and on the role of epicardial and visceral adipose tissue. We conclude by discussing recent evidence suggesting that several therapeutic strategies may reverse this pro-arrhythmic milieu, with a particular focus on established anti-obesity treatments and emerging experimental therapeutic approaches.

## 1. Introduction

Obesity has reached epidemic proportions on a global scale, with a steadily increasing prevalence, particularly in high-income countries [1]. Its impact extends far beyond traditional cardiometabolic complications, encompassing a broad spectrum of cardiovascular (CV) disorders. Among these, cardiac arrhythmias—particularly atrial fibrillation (AF)—constitute a major and growing contributor to morbidity, healthcare utilization, and impaired quality of life in obese patients. AF is now the most common sustained arrhythmia globally, and its increasing incidence parallels the rising prevalence of obesity, suggesting a close and potentially causal relationship between these two conditions [2].

Historically, obesity has been regarded primarily as a comorbidity that exacerbates established CV disease. However, accumulating evidence supports a paradigm shift: obesity should be recognized as a potential CV risk factor and an active arrhythmogenic substrate. Excess adiposity promotes structural remodeling, including atrial and ventricular dilation, left ventricular (LV) hypertrophy, and myocardial fibrosis. In parallel, electrical alterations mediated by inflammation, autonomic imbalance, metabolic dysfunction, and adipose tissue infiltration of the myocardium contribute to the pro-arrhythmogenic milieu. These processes create a vulnerable substrate that facilitates both the initiation and maintenance of arrhythmias and possibly a different response to anti-arrhythmogenic therapies. Importantly, conventional arrhythmia management strategies—such as pharmacological rhythm control, electrical cardioversion, and catheter ablation—often demonstrate reduced efficacy in individuals with obesity (especially with BMI > 40 kg/m^2^) when not accompanied by targeted metabolic interventions [3]. This underscores the limitations of a purely electrophysiological approach that fails to address the underlying substrate.

In this context, the thesis of this narrative review is that obesity (particularly adipose tissue remodeling) is a driving force of cardiac structural and electrical remodeling, directly contributing to arrhythmogenesis, and that this pro-arrhythmic milieu may be at least partially reversible through comprehensive weight and metabolic management. For clarity and conciseness, we will focus on the most common clinically relevant arrhythmias and on the pharmacological and non-pharmacological interventions, suggesting or supporting a reduction in arrhythmic risk.

## 2. From Adiposity to Adipose Tissue Dysfunction and Remodeling

Available evidence suggests that the central pathological link between obesity and CV disease lies not simply in the accumulation of adipose tissue but in the progressive remodeling and dysfunction of adipose depots. Overnutrition promotes adipose tissue expansion, which leads to a local inflammatory response. Although the initial inflammatory trigger remains incompletely understood, it has been proposed that early adipose tissue inflammation may represent an adaptive response to tissue expansion and the increased metabolic demands associated with nutrient excess. However, when these stimuli exceed the tissue’s adaptive capacity, this response may become chronic and maladaptive, contributing to a state of metabolic inflexibility characterized by impaired insulin signaling, altered lipid metabolism, and reduced responsiveness to catecholamines. In a recent study, Miranda et al. demonstrated that obesity is associated with extensive remodeling of the adipose niche, involving adipocytes, progenitor cells, vascular cells, and immune populations, and characterized by adipocyte hypertrophy, increased inflammatory cell infiltration, cellular senescence, and alterations in extracellular matrix (ECM) and vascular compartments [4]. Thus, adipose tissue remodeling may translate into systemic metabolic and CV consequences, making adipose tissue an active endocrine and paracrine organ. As adipose tissue expands beyond its adaptive capacity, particularly within visceral depots, alterations in adipocyte function and tissue inflammation can modify the production and release of adipokines, cytokines, and other bioactive mediators, which may act locally or enter the circulation. In this context, the adipose tissue expandability hypothesis proposed by Virtue and Vidal-Puig suggests that the failure of adipose tissue to appropriately accommodate excess energy shifts its role from a protective lipid-buffering compartment towards a source of metabolic and inflammatory signals capable of contributing to systemic disease [5].

## 3. Adipose Tissue Remodeling and Potential Arrhythmogenic Effects

### 3.1. Visceral Adipose Tissue (VAT)

In non-obese individuals, visceral adipose tissue (VAT) is an endocrine organ that stores excess lipids, limiting ectopic fat deposition, and contributing to metabolic regulation through the secretion of adipokines that support insulin sensitivity and vascular homeostasis. In this balanced state, VAT maintains an anti-inflammatory profile, with limited immune activation and controlled cytokine release.

In obesity, VAT undergoes expansion and functional dysregulation, shifting to a driver of cardiometabolic disease. Hypertrophic adipocytes become hypoxic and insulin-resistant, leading to increased lipolysis and excessive release of free fatty acids into the circulation. This is associated with immune cell infiltration (M1 macrophages) and a pro-inflammatory shift in adipokine secretion, characterized by elevated levels of cytokines such as TNF-α and IL-6. As a result, VAT promotes chronic low-grade systemic inflammation, endothelial dysfunction, and ectopic lipid deposition in peripheral organs, including the myocardium [6]. These alterations contribute to insulin resistance, metabolic syndrome, and increased CV risk, positioning VAT as a key mediator of obesity-related complications.

Supplementary Table S1 summarizes the main molecular mechanisms potentially linking VAT to arrhythmogenesis in clinical and preclinical studies only involving overweight or obese subjects.

**Figure 1:**
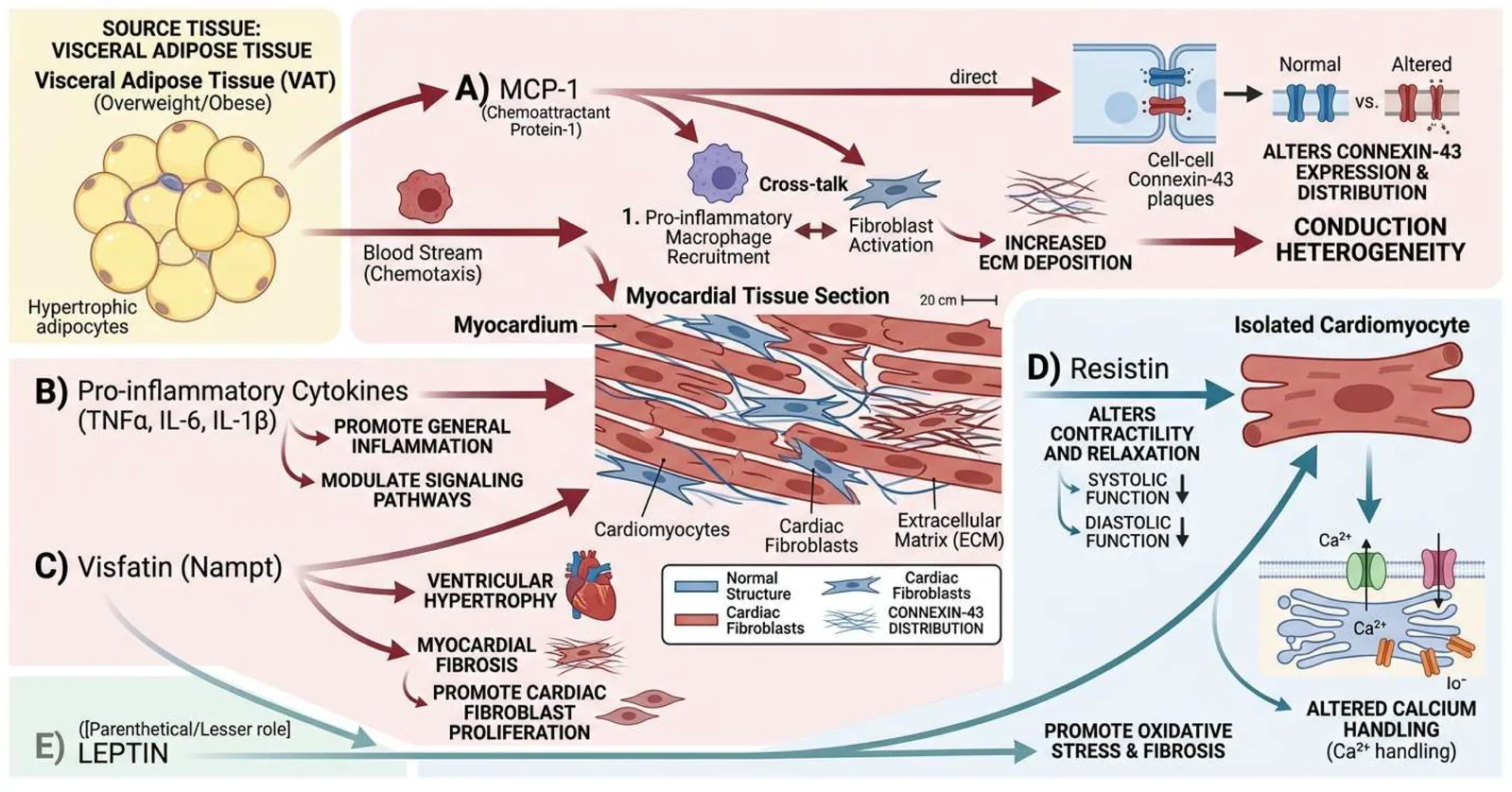
Mechanistic pathways linking visceral adipose tissue secretion and potential pro-arrhythmogenic remodeling. The illustrated mechanisms predominantly derive from preclinical studies. Leptin has been included for completeness despite less conclusive evidence regarding its primary production by VAT compared to other adipose depots. VAT, Visceral Adipose Tissue; MCP-1, Monocyte Chemoattractant Protein-1; TNFα, Tumor Necrosis Factor-Alpha; IL-6, Interleukin-6; IL1β, Interleukin-1 beta; Nampt, Nicotinamide Phosphoribosyltransferase (Visfatin); ECM, Extracellular Matrix; and Cx43, Connexin-43. Image created with Gemini AI (3.6 Flash 2026).

Based largely on indirect evidence, ex vivo, in vitro studies, and preclinical models, VAT secretory activity can promote myocardial tissue alterations (Figure 1). Specifically, VAT from overweight or obese individuals secretes high levels of monocyte chemoattractant protein-1 (MCP-1), inflammatory cytokines (such as TNF-α, IL-6, and IL-1β), visfatin, and resistin. MCP-1 (CCL2) promotes the recruitment of circulating monocytes into the myocardium, leading to the accumulation of pro-inflammatory macrophages and the induction of myocardial fibrosis [7]. Furthermore, the inflammatory milieu driven by MCP-1 may indirectly induce electrical remodeling by altering the expression of ion channels and gap junction proteins—particularly connexin-43—resulting in increased conduction heterogeneity. Accordingly, elevated MCP-1 levels have been linked to AF in several clinical studies [8,9], while experimental models have implicated MCP-1 signaling in myocardial inflammation, fibrosis, and adverse electrical remodeling [10].

Resistin is a pro-inflammatory adipokine primarily secreted by adipose tissue immune cells, particularly macrophages. Preclinical studies suggest that resistin impairs cardiomyocyte contractility and relaxation while altering intracellular calcium handling [11]. Circulating resistin levels are elevated in obesity, with evidence indicating enhanced resistin expression in both VAT and subcutaneous adipose tissue (SAT) [12]. Growing evidence supports a potential role for resistin in arrhythmogenesis, particularly in AF. Elevated resistin concentrations have been associated with an increased risk of incident AF during long-term follow-up (7.6–11 years) [13,14]. Moreover, resistin levels correlate with AF phenotype, disease progression [15], and recurrence following catheter ablation [16], suggesting that resistin may contribute to the development and maintenance of an arrhythmogenic substrate. Interestingly, large Mendelian randomization studies link resistin levels to AF risk, but with a relationship likely mediated by the presence of hypertension [17].

Leptin is a pleiotropic adipokine predominantly secreted by white adipose tissue, with circulating levels proportional to adiposity [12]. Experimental studies show that chronic hyperleptinemia, particularly in obesity, is associated with systemic hypertension, potentially through increased sympathetic activity and vascular remodeling [18,19]. At the myocardial level, leptin signaling activates pro-hypertrophic pathways, including JAK2/STAT3, MAPK, PI3K/Akt/mTOR, and calcineurin/NFAT, while chronic hyperleptinemia may impair mitochondrial homeostasis, promoting oxidative stress, cardiomyocyte hypertrophy, fibrosis, and adverse ventricular remodeling [20,21]. Importantly, leptin may also directly modulate atrial electrophysiology: in isolated rabbit atrial myocytes, leptin altered action-potential duration, ionic currents, and intracellular Ca^2+^ handling, demonstrating a direct electrophysiological effect on atrial cardiomyocytes [22]. Despite these findings, their direct translational relevance to clinical practice remains to be established. Also, several critical aspects remain to be elucidated, including its dose–response relationship and the differential cardiac effects of acute versus chronic hyperleptinemia. Notably, leptin is predominantly synthesized and secreted by SAT rather than VAT, adding a layer of complexity to its distinct contribution to adipose-mediated arrhythmogenesis. Indeed, clinical evidence linking circulating leptin to AF remains less consistent: in a large cohort of postmenopausal women, leptin levels were not independently associated with incident AF after multivariable adjustment, unlike resistin levels [14].

Visfatin, also known as nicotinamide phosphoribosyltransferase (NAMPT), is a multifunctional adipokine predominantly secreted by VAT, but also expressed by cardiomyocytes, macrophages, and endothelial cells. Elevated circulating visfatin levels have been associated with obesity, insulin resistance, metabolic syndrome, and cardiovascular disease [23]. Preclinical evidence suggests that visfatin/NAMPT may contribute to obesity-related cardiac remodeling: cardiac overexpression of visfatin/NAMPT in mice induced pathological ventricular hypertrophy and fibrosis via activation of the MAPK and calcineurin/NFAT signaling pathways, generating structural alterations that may favor an arrhythmogenic substrate [24]. In addition, visfatin directly promotes cardiac fibroblast proliferation and collagen type I/III synthesis through PI3K-, ERK1/2-, and p38 MAPK-dependent pathways, further supporting its potential role in myocardial fibrotic remodeling [25]. However, direct evidence linking visfatin to arrhythmias in humans remains lacking.

In the setting of obesity and metabolic dysregulation, expanded and remodeled VAT undergoes marked cellular stress characterized by adipocyte hypertrophy, tissue hypoxia, and local inflammation, which significantly accelerates the release of EVs (VAT-EVs) into the systemic circulation. In obesity, systemic circulating VAT-EVs can reach the cardiac tissue and deliver non-coding RNAs, signaling proteins, and lipid molecules, potentially influencing cardiac remodeling. A recent preclinical work by Limpitikul et al. demonstrated that exposure to obese VAT-EVs induces action potential duration prolongation, repolarization instability, and impaired calcium handling in human atrial myocytes and iPSC-derived cardiomyocytes in vitro [26]. Mechanistically, VAT-EVs upregulate the TRPC3 channel, increasing cation influx, while pharmacological TRPC3 blockade normalized these electrophysiological abnormalities. Furthermore, in cellular models, VAT-EVs actively remodel the interstitial niche by driving fibroblast-to-myofibroblast differentiation (causing ECM/collagen accumulation) and shifting cardiac macrophages toward a pro-inflammatory phenotype. While transcriptomic-wide association studies (TWAS) link these cellular responses to human genomic loci, these findings remain strictly preclinical, and dedicated clinical studies evaluating the direct role of EVs in obesity-related arrhythmogenesis in humans are currently lacking [27]. Gene pathways dynamically regulated by VAT-EV cargo overlap significantly with polymorphisms associated with clinical AF susceptibility and QT interval prolongation in large-scale human Genome-Wide Association Study (GWAS) datasets, suggesting a genetic pathway linking VAT to arrhythmia risk.

In synthesis, evidence linking the secretory activity of VAT to arrhythmogenesis remains largely indirect, with much of the available evidence deriving from ex vivo and in vitro experimental studies. Although these studies have provided important insights into the potential effects of adipose-derived mediators on cardiac electrophysiology and tissue remodeling, their findings do not necessarily establish that these mechanisms operate to the same extent in vivo or translate into clinically relevant arrhythmogenesis in humans. Also, as observed with epicardial adipose tissue (EAT, see below), the precise timeline and magnitude of VAT-driven arrhythmogenic remodeling, as well as its independent contribution relative to general obesity, remain unclarified. Prospective interventional studies directly targeting VAT and mechanistic trials linking distinct adipo-secretome profiles to arrhythmic outcomes are needed to confirm these findings.

### 3.2. Epicardial Adipose Tissue (EAT)

Epicardial adipose tissue (EAT) is a fat depot located between the myocardial surface and the visceral layer of the pericardium, supplied by branches of the coronary artery. It shares the coronary microcirculation with the underlying myocardium, enabling direct paracrine and vasocrine interactions. Histologically, EAT is composed of small adipocytes, stromal-vascular cells, and resident inflammatory cells such as lymphocytes (CD3+), macrophages (CD68+), and mast cells. Furthermore, ganglionated plexi (GPs) from the autonomic nervous system are also located in the epicardial fat pads and modulate cardiac autonomic input [28].

**Figure 2:**
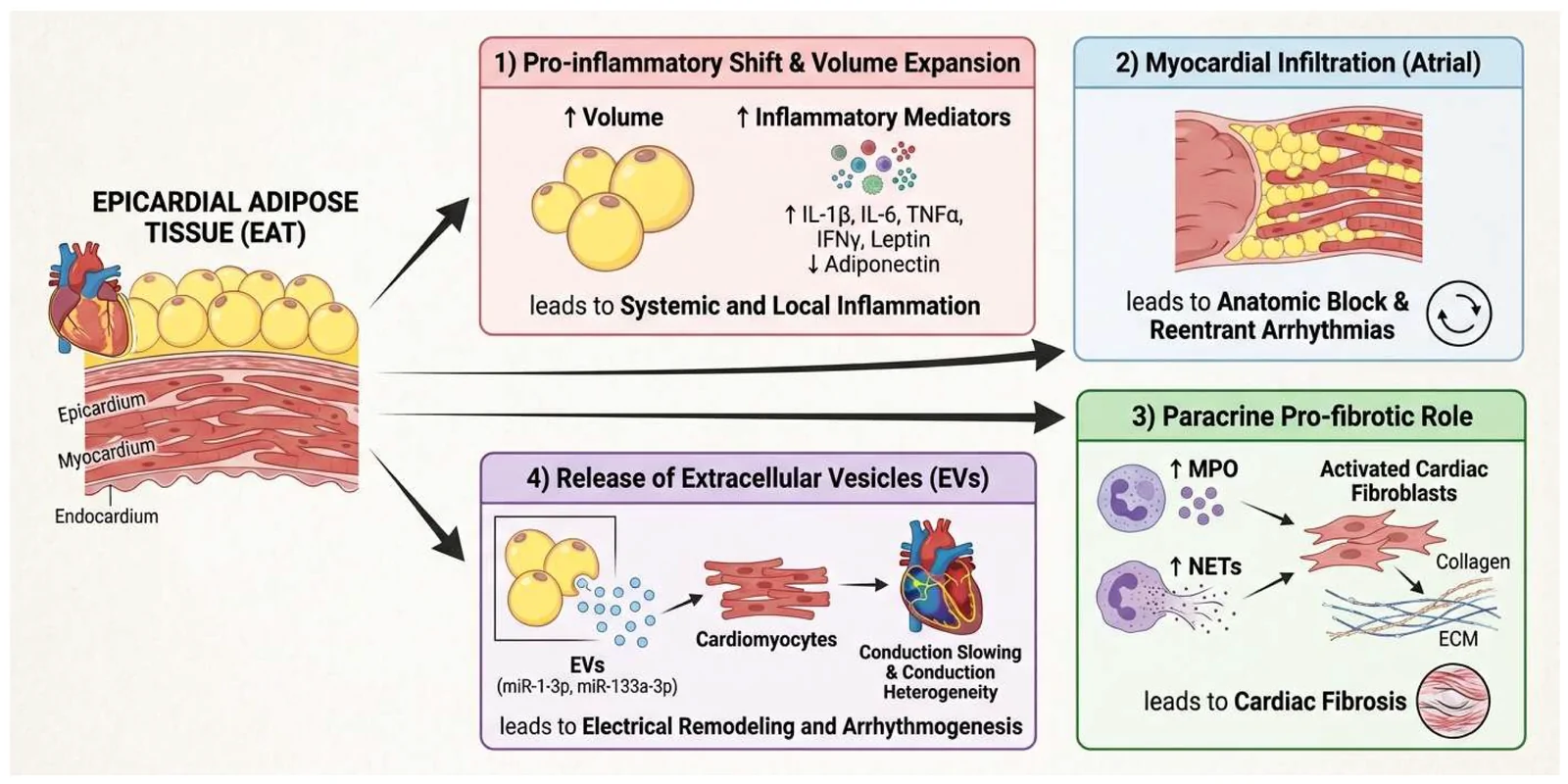
Mechanistic pathways linking epicardial adipose tissue secretion and potential pro-arrhythmogenic remodeling. The illustrated mechanisms predominantly derive from preclinical studies. EAT, Epicardial Adipose Tissue; ECM, Extracellular Matrix; EVs, Extracellular Vesicles; IFNγ, Interferon-gamma; IL-1β, Interleukin-1 beta; IL-6, Interleukin-6; miR/miRNA, MicroRNA; MPO, Myeloperoxidase; NETs, Neutrophil Extracellular Traps; TNFα, Tumor Necrosis Factor-alpha. Image created with Gemini AI (3.6 Flash 2026).

Under physiological conditions, EAT serves as a cardioprotective energy reservoir, providing fatty acids to the myocardium, contributing to mechanical and thermal protection, and secreting anti-inflammatory adipokines such as adiponectin. In obesity, EAT may undergo a phenotypic shift toward a pro-inflammatory and pro-fibrotic state, potentially contributing to myocardial remodeling and arrhythmogenesis through paracrine and vasocrine signaling (Figure 2). While early research focused primarily on soluble adipokines and inflammatory cytokines, extracellular vesicles (EVs), including exosomes and microvesicles, have emerged as crucial mediators of the EAT-myocardial cross-talk.

Although EAT expansion is consistently associated with increasing adiposity, studies specifically investigating EAT in exclusively obese populations remain relatively limited, with many human studies including mixed populations of overweight and obese individuals or patients with concomitant CV disease. In a human study of patients undergoing cardiac surgery, Vyas et al. showed that overweight and obesity were associated with changes in the inflammatory and metabolic profile of EAT, including increased expression of IL-1, IL-6, TNF-α, IFN-γ, and leptin, together with extensive transcriptional remodeling of inflammatory and metabolic pathways [29]. Consistent with these findings, Cabaro et al. showed that EAT-derived IL-1β secretion was higher in overweight patients developing postoperative AF (mean BMI 29.4 kg/m^2^) and that IL-1β and IL-18 promoted human atrial fibroblast proliferation and collagen production in vitro, suggesting a potential link between EAT inflammation and atrial fibrotic remodeling [30].

More broadly, in obesity, the expansion and functional remodeling of EAT may represent an important link between excess adiposity and CV disease, including coronary artery disease and cardiac arrhythmias. Packer proposed that EAT acts as a local transducer of systemic metabolic and inflammatory stress; this phenotypic transformation alters adipokine secretion, thereby driving myocardial inflammation, microvascular dysfunction, fibrotic and electrical remodeling, atrial arrhythmogenesis, coronary atherosclerosis, and HFpEF pathogenesis [31].

EAT appears to be a key mediator in the association between AF and obesity, potentially being more important than BMI or visceral adiposity. Indeed, the EAT secretome from overweight patients with AF downregulates connexin43 expression in atrial cardiomyocytes, with impairment in electrical coupling, slowed conduction velocity, and enhanced spatial conduction heterogeneity [32]. Besides EAT remodeling towards a pro-inflammatory, lipotoxic organ, preclinical models suggest that EAT can infiltrate the atrial myocardium, particularly in the posterior LA and around the pulmonary veins, where it facilitates reentry circuits [26,33]. Available data also indicate a pro-fibrotic role of EAT in patients with AF through the expression of myeloperoxidase (MPO) and increased presence of neutrophil extracellular traps (NETs) [34], suggesting the induction of atrial fibrosis. However, direct evidence specifically demonstrating obesity-driven EAT accumulation in humans remains limited, and most current findings derive from ex vivo and in vitro studies.

In the context of obesity-induced chronic inflammation, EAT releases EVs at a higher rate than SAT. Unhindered by anatomical barriers, these EAT-EVs deliver pro-inflammatory signals directly to adjacent cardiomyocytes and fibroblasts. Mechanistically, in vitro data show that hypertrophic adipocyte-derived EVs carry elevated miR-802-5p, which downregulates HSP60 in cardiomyocytes. This disrupts mitochondrial homeostasis, increases reactive oxygen species (ROS) production, and promotes cardiac insulin resistance, ultimately destabilizing myocyte homeostasis and predisposing the myocardium to arrhythmogenesis [35]. EAT-derived EVs from overweight individuals are enriched in miR-1-3p and miR-133a-3p. Overexpression of these miRNAs in cardiomyocytes suppresses the expression of KCNJ2 and KCNJ12, respectively, causing conduction slowing and increased conduction heterogeneity [36]. Beyond electrophysiological changes, EAT-derived EVs can promote structural remodeling. Indeed, EAT-derived EVs isolated from overweight patients with AF contain a distinct proteomic and miRNA profile enriched with pro-inflammatory and pro-fibrotic factors [37]. When internalized by atrial fibroblasts and cardiomyocytes, these EVs induce cellular hypertrophy, promote myofibroblast differentiation, and stimulate excessive ECM collagen deposition. In line with these findings, circulating EVs from obese patients with AF displayed a profibrotic miRNA profile, with increased miR-146b and decreased miR-133a and miR-29a. In this context, miR-146b reduces TIMP-4 expression and is associated with increased MMP-9 activity and ECM remodeling [38].

In contrast to AF, evidence linking EAT to ventricular arrhythmogenesis is still limited, particularly in obesity. Experimental and observational studies suggest that both the amount and regional distribution of EAT, especially over the RV, may contribute to ventricular electrical heterogeneity. However, most available data derive from experimental models or patients with established cardiac disease rather than obese individuals, and a direct causal relationship between obesity-related EAT remodeling and ventricular arrhythmias (VAs) remains to be demonstrated.

Taken together, these data indicate a possible paracrine role of EAT in atrial structural and functional remodeling, thereby favoring arrhythmogenesis. However, current evidence is largely derived from observational human studies and preclinical models and does not adequately capture EAT remodeling and its secretome changes as a function of the presence and degree of visceral adiposity. Future prospective interventional trials targeting EAT directly are needed to definitively establish causality and disentangle its depot-specific mechanisms from systemic obesity.

Supplementary Table S2 summarizes the most recent clinical and translational evidence focused on overweight/obesity and EAT paracrine function.

## 4. From Adipose Signaling to Structural Cardiac Remodeling in Obesity

### 4.1. Left Atrial Remodeling

Obesity drives LA structural and functional remodeling through a combination of volume overload (increased blood volume and cardiac output) and pressure overload (elevated LV filling pressures). This leads to LA enlargement—a key marker of AF and stroke risk—which begins early in life and is exacerbated by coexisting hypertension [39,40]. Functionally, severe obesity impairs all LA strain components (reservoir, conduit, and contractile phases) [41]. Although mild-to-moderate obesity may transiently boost LA contractile function as a compensatory response to preserve cardiac output, this adaptation deteriorates as atrial dysfunction advances, leading to progressive LA remodeling [42].

Obesity promotes atrial arrhythmogenesis through structural, electrical, and functional alterations within the atria. Studies on animal models of obesity (mainly ovine models) first showed increased LA volume, fibrosis, inflammatory infiltration, and lipidosis, associated with progressive conduction slowing and an increase in regional conduction heterogeneity with obesity, especially in zones adjacent to epicardial fat depots [42,43]. Mahajan et al. performed electroanatomical mapping and cardiac magnetic resonance in patients undergoing AF ablation and divided them into two groups based on BMI (<27 kg/m^2^ or ≥27 kg/m^2^). The obese group (mean BMI 30.2 kg/m^2^) showed reduced LA conduction velocity, increased electrogram fractioning, and a significant augmentation in low-voltage areas within the atria, especially in zones adjacent to EAT (posterior and inferior LA wall). Interestingly, obese patients also displayed increased EAT measurements (LA EAT volume, ventricular EAT volume, and total EAT volume), which correlated better with LA conduction abnormalities than BMI [44]. Further studies conducted on patients without AF undergoing coronary bypass surgery (mean BMI 30 kg/m^2^) show that fibrotic areas within the atria correlate with local EAT depots (in this case the anterior right atrium depots) and lead to local conduction slowing; zones with local EAT depots are characterized by increased adipose tissue infiltration within the myocardium as well as the redistribution of gap junction proteins (such as connexin 40, Cx40) in cardiomyocytes. In vitro assays also demonstrate a paracrine effect of EAT, inducing a decreased spontaneous beating rate and a prolonged action potential duration [45].

These findings suggest that, in obesity, atrial arrhythmogenic mechanisms may be driven by the presence of localized fibrosis, slowing and fractionation of electrical conduction, and the deleterious effects of EAT. The latter promotes fatty infiltration of the atrial myocardium, along with paracrine pro-inflammatory and pro-fibrotic effects (see Figure 2).

### 4.2. Left Ventricular Remodeling

Obesity drives LV structural and functional remodeling through a dual mechanism of pressure overload (hypertension, arterial stiffness) and volume overload (expanded blood volume, high cardiac output). This leads to increased LV mass and geometry alterations—predominantly eccentric hypertrophy, even if concentric remodeling also occurs. Systemic inflammation, oxidative stress, and Renin–Angiotensin–Aldosterone System (RAAS) activation further promote myocardial fibrosis and ECM deposition. Consequently, LV hypertrophy is highly prevalent in obesity (affecting over 50% of individuals) [46], where increasing BMI and central adiposity directly correlate with impaired LV relaxation, elevated filling pressures (E/E′), and progressive diastolic dysfunction, even in the absence of overt systolic impairment [47,48].

In obesity, a complex interplay between cellular mechanisms and mechanical forces contributes to adverse LV remodeling. Besides mechanical stress and both volume and pressure overload, obesity activates the RAAS, contributing to cardiomyocyte hypertrophy, oxidative stress, and fibroblast activation [49]. Obesity-associated chronic low-grade inflammation further promotes the release of pro-inflammatory cytokines (TNF-α, IL-6, and IL-1β) and activates the NADPH-like/NOD-like Leucine-rich Repeat Protein 3 (NLRP3) inflammasome, generating a pro-fibrotic environment that increases LV stiffness [50]. Dysregulated adipokine signaling, characterized by increased leptin and resistin levels and reduced adiponectin, further amplifies these remodeling pathways. Finally, obesity-related insulin resistance induces impaired myocardial glucose uptake, metabolic inefficiency, and increased reactive oxygen species production, contributing to progressive structural and functional cardiac impairment [51].

Recent evidence highlights myocardial fibrosis as a central mechanism linking obesity to structural and functional LV impairment. The above-mentioned mechanisms indeed can promote ECM accumulation, ventricular stiffening, and the development of obesity-related heart failure, particularly HFpEF [49]. A prospective CMR study of adults with metabolically healthy obesity found a greater prevalence of myocardial fibrosis compared with non-obese healthy controls, with fibrosis detected in 44% of metabolically healthy and 71% of metabolically unhealthy individuals, despite preserved LV ejection fraction. Also, higher BMI was independently associated with myocardial fibrosis, suggesting that myocardial tissue remodeling may occur even in the absence of overt metabolic disease [52]. Several interconnected inflammatory, adipokine, and neurohormonal pathways have been implicated in obesity-associated myocardial fibrosis. Preclinical and clinical evidence points to activation of the NLRP3 inflammasome and its downstream cytokines, particularly IL-1β and IL-18, as potential drivers of fibrotic remodeling [53]. Adipokines may also contribute, with leptin promoting cardiac fibroblast activation and ECM deposition through inflammatory, oxidative, and profibrotic pathways involving TGF-β, the RAAS, endothelin-1, and mineralocorticoid receptor signaling. More recently, PCPE-1 and locally produced cardiac thyrotropin-releasing hormone (TRH) have emerged as potential mediators of obesity-related myocardial fibrosis and diastolic dysfunction, although evidence for these pathways remains largely preclinical [54,55].

### 4.3. Right Ventricular Remodeling

Historically, the association between obesity and right ventricular (RV) dysfunction was largely attributed to the high prevalence of obstructive sleep apnea syndrome (OSAS), pulmonary hypertension, or pulmonary restrictive syndrome in this population. However, several studies have shown that RV dysfunction in obesity occurs independently of sleep-disordered breathing or pulmonary hypertension. Rather, it is likely driven by a combination of interrelated mechanisms, including chronic hemodynamic overload and intrinsic cardiac remodeling. Wong et al. showed that obesity is associated with subclinical RV dysfunction in individuals without overt CV disease: using echocardiography, they showed that increasing BMI correlates with progressive impairment of RV systolic performance, alterations that were independent of the presence of OSAS and its severity [56].

The presence of RV remodeling in obese individuals in the absence of overt CV disease has also been described by Rider et al. In a large cohort of 739 healthy individuals spanning a wide BMI range (15.3 to 59.2 kg/m^2^), they showed that obesity is associated with significant RV remodeling in the absence of traditional CV risk factors. RV mass increased progressively with BMI in both sexes, indicating obesity-related RV hypertrophy. Interestingly, sex-specific differences in remodeling patterns were observed: concentric RV remodeling was more pronounced in men. Also, greater RV mass and RV concentric remodeling were independently associated with impaired LV diastolic filling, suggesting a potential adverse interaction between obesity-related RV structural changes and global cardiac function [57]. These findings highlight sex-dependent patterns of RV adaptation in obesity, characterized by a predominantly hypertrophic response in men and a combined hypertrophic–dilatory phenotype in women. Using CMR, Lewis et al. investigated the impact of obesity on RV structure, function, and hemodynamic response. They demonstrated that obesity is associated with adverse RV remodeling, characterized by increased RV mass and evidence of functional impairment, alongside altered ventricular response to physiological stress. They also confirmed that these changes occur even in the absence of overt CV disease, supporting the presence of early, obesity-related myocardial remodeling [58].

Besides subclinical RV dysfunction in otherwise healthy individuals with obesity, RV dysfunction may frequently result from increased RV afterload. This may be driven by increased pulmonary vascular resistance associated with OSAS and obesity hypoventilation syndrome (OHS) through recurrent hypoxemia, hypercapnia, sympathetic activation, endothelial dysfunction, and hypoxic pulmonary vasoconstriction, which may promote pulmonary vascular remodeling. Obesity-related LV diastolic dysfunction may further increase left-sided filling pressures and cause post-capillary pulmonary hypertension, thereby increasing RV afterload. Increased circulating blood volume and cardiac output may additionally impose a chronic volume load on the RV, while negative intrathoracic pressure during obstructive events may acutely increase RV preload. Finally, several mechanisms already described for LV remodeling—including neurohormonal activation, insulin resistance, lipotoxicity, oxidative stress, inflammation, and myocardial fibrosis—may also contribute to adverse RV remodeling and dysfunction.

## 5. Adipose-Driven Electropathy

**Figure 3:**
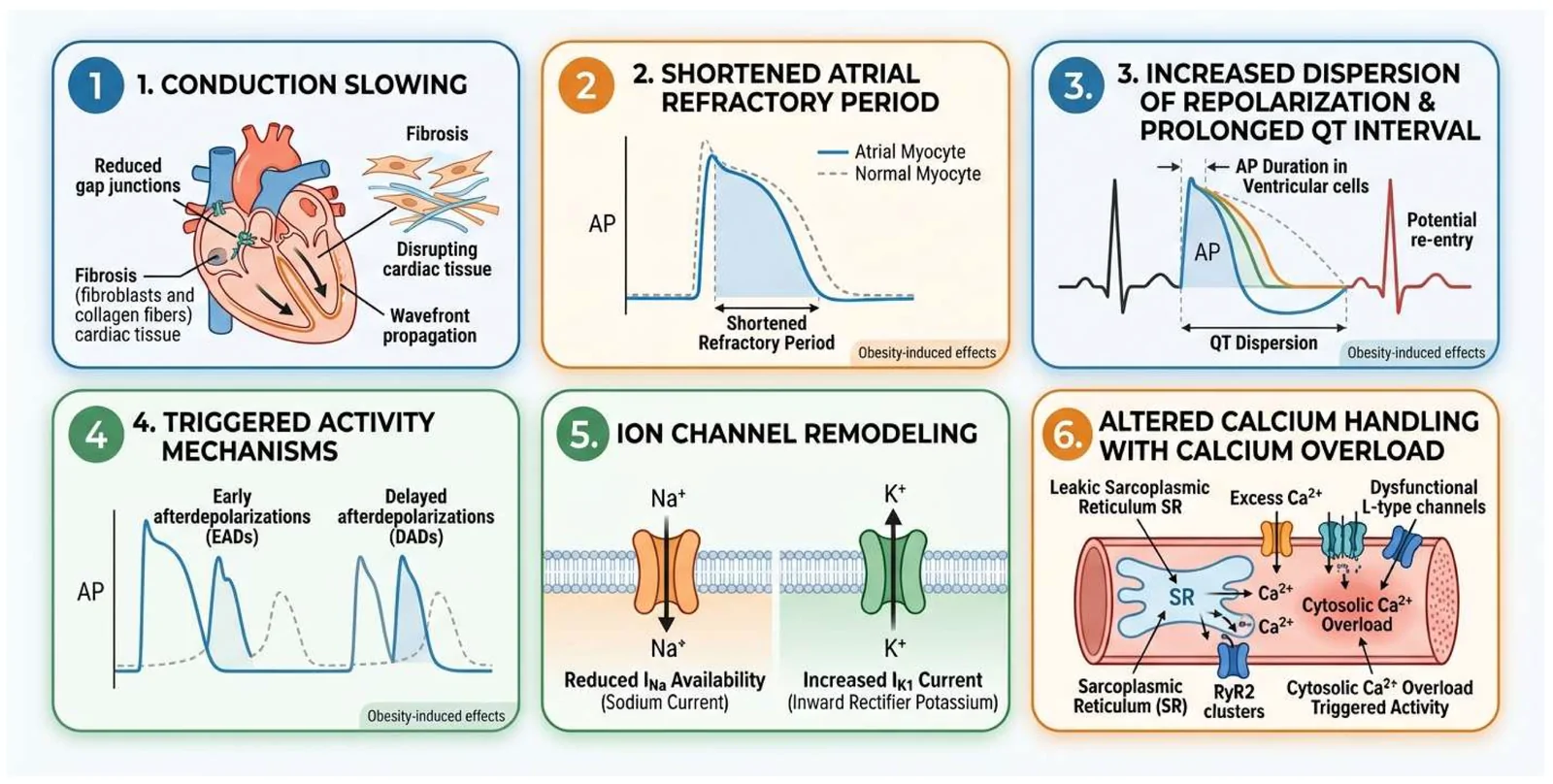
Summary of the mechanisms involved in adipose-driven electropathy. The illustrated mechanisms derive from both preclinical studies and studies on humans. AP, Action Potential; SR, Sarcoplasmic Reticulum; RyR2, Ryanodine Receptor 2. Image created with Gemini AI (3.6 Flash 2026).

Obesity induces a complex electrophysiological remodeling that significantly contributes to arrhythmogenesis. This process results from the interplay between structural remodeling, metabolic dysfunction, and direct cellular electrophysiological changes mediated by adipose tissue, ultimately creating a substrate that favors both re-entry and abnormal impulse generation. Several electrical alterations are at play described below and summarized in Figure 3.

### 5.1. Conduction Slowing

Conduction slowing represents a central feature of atrial remodeling in obesity. It is primarily driven by structural disruption of myocardial architecture infiltration caused by EAT and interstitial fibrosis, which acts as both a physical and electrical barrier to impulse propagation. In addition to structural discontinuities, EAT exerts paracrine effects that impair intercellular electrical coupling, including alterations in connexin expression and distribution. Clinical and experimental data demonstrate that increased local EAT volume is associated with reduced conduction velocity, electrogram fractionation, and increased conduction heterogeneity [59,60]. Greater voltage heterogeneity and increased low-voltage areas assessed by endocardial mapping are reported in the obese group: these changes facilitate conduction block and promote the formation of reentrant circuits [26]. Conduction slowing assessed by 12-lead electrocardiogram is also evident, as demonstrated by progressive prolongation of the PR interval, maximum P-wave duration, and interatrial conduction indices with increasing BMI and epicardial fat burden, reflecting worsening atrial conduction with increasing adiposity [61,62,63].

### 5.2. Shortened Atrial Refractory Period

Shortening of the atrial effective refractory period (AERP) has been reported in obesity-related electrophysiological remodeling. In clinical electrophysiological studies, obese patients demonstrated shorter effective refractory periods in the left atrium (233 ± 32 ms vs. 251 ± 25 ms), as well as in the proximal (207 ± 33 ms vs. 248 ± 34 ms) and distal pulmonary veins (193 ± 33 ms vs. 248 ± 44 ms), compared with individuals with normal BMI [64]. This alteration may promote both the initiation and maintenance of AF. Mechanistically, these changes are likely driven by a combination of ion channel remodeling and autonomic imbalance. Dysfunctional or excessive EAT accumulation can lead to increased activity of ganglionated plexi, resulting in both sympathetic and parasympathetic stimulation. Sympathetic nerve activation has been shown to increase calcium transients within myocytes, while parasympathetic activation causes a reduction in the atrial refractory period and thereby facilitates reentry, both contributing to an AF substrate [65].

### 5.3. Increased Dispersion of Repolarization

Obesity is also associated with increased spatial dispersion of repolarization, which creates electrophysiological heterogeneity across the atrial myocardium. This heterogeneity provides the substrate for unidirectional block and wavebreak, both essential for the initiation and maintenance of reentrant arrhythmias. As reported above, regional differences in fibrosis, adipose tissue distribution, and ion channel expression underlie this dispersion. In particular, exposure of cardiomyocytes to adipokines such as tumor necrosis factor-α (TNF-α), interleukin-6 (IL-6), and IL-1β has been shown to induce cytokine-mediated changes in ionic currents that lead to action potential duration (APD) prolongation, thereby contributing to regional heterogeneity of repolarization [65,66].

Importantly, these alterations are not limited to the atria but extend to the ventricular myocardium, where repolarization abnormalities may manifest as QT interval prolongation. Clinical studies have shown that obesity is associated with increased QTc duration (as measured by electrocardiography), a well-established marker of repolarization heterogeneity and electrical instability. In obesity, increased adipose tissue leads to abnormally high expression of the aromatase enzyme, which converts testosterone to estrogens. Whereas testosterone shortens the action potential, estrogens prolong the QTc interval by reducing the myocardial potassium channel current. The effect of adipose tissue on QTc lengthening is also supported by prior studies that have shown that QTc duration shortens significantly in response to weight loss after bariatric surgery [67]. In this context, increased dispersion of repolarization represents a possible mechanistic link between obesity and the risk of SCD, particularly in the presence of additional triggers such as ischemia, autonomic imbalance, or electrolyte disturbances [59].

### 5.4. Triggered Activity Mechanisms

In addition to re-entry, obesity promotes triggered activity through both early and delayed afterdepolarizations. These mechanisms are closely linked to metabolic and oxidative stress. Inflammatory adipokines secreted by epicardial adipose tissue have been shown to induce intracellular calcium overload by reducing SERCA2a activity and altering the expression and function of key calcium-handling proteins. This promotes activation of depolarizing currents via the sodium–calcium exchanger, favoring delayed afterdepolarizations, thereby contributing to arrhythmogenesis [43,66,68]. Moreover, inflammatory adipokines seem to regulate L-type calcium channels and to reduce transient outward potassium repolarizing currents (Ito) in preclinical studies, prolonging the atrial action potential duration and promoting early afterdepolarizations [44,45,66].

### 5.5. Ion Channel Remodeling

EAT secretome, especially adipokines such as tumor necrosis factor-α (TNF-α), interleukin-6 (IL-6), and IL-1β, exerts a paracrine effect on the myocardium by modulating ion channel function and calcium handling. This results in APD prolongation and increased spatial heterogeneity of repolarization, thereby facilitating re-entry mechanisms in the myocardium. Moreover, abnormal depolarizing currents can cause early and delayed depolarizations, ultimately promoting ectopic beats and arrhythmogenesis [65,69].

### 5.6. Sodium Channel Changes

Reduced sodium current (INa) availability leads to decreased excitability and contributes to conduction slowing. This effect is likely mediated by metabolic stress, inflammation, and structural remodeling, including impaired gap junction coupling. Experimental data suggest that adipose tissue–derived factors and oxidative stress can alter sodium channel function and intercellular electrical coupling, further contributing to conduction abnormalities [59,66].

### 5.7. Potassium Currents

Alterations in potassium currents play a central role in obesity-related electrophysiological remodeling. Increased inward rectifier potassium current (IK1) and changes in repolarizing currents contribute to shortening of the action potential duration (APD) and atrial refractoriness, thereby stabilizing reentrant circuits. These changes may be driven by metabolic derangements, autonomic imbalance, and adipose tissue–derived signaling pathways [69,70].

### 5.8. Calcium Handling Abnormalities

Calcium handling is profoundly altered in obesity. Lipotoxicity and mitochondrial dysfunction impair intracellular calcium homeostasis, leading to calcium overload and spontaneous sarcoplasmic reticulum calcium release. This promotes delayed afterdepolarizations via activation of the sodium–calcium exchanger.

In addition, increased reactive oxygen species (ROS) production further destabilizes calcium cycling and may facilitate early afterdepolarizations. These abnormalities represent key mechanisms linking metabolic dysfunction to triggered activity and arrhythmogenesis [66,70].

## 6. Epidemiology of Arrhythmias in Obesity

### 6.1. Atrial Fibrillation in Obesity

Atrial fibrillation (AF) and obesity are strongly associated, with robust evidence supporting a dose–response relationship between BMI and AF incidence: the risk of developing AF increases progressively with rising BMI [71]. Measures of both general adiposity (BMI) and visceral adiposity (waist circumference, waist-to-hip ratio) closely correlate with increased AF risk, underscoring the pathophysiological relevance of visceral adiposity [2]. Indeed, BMI does not adequately capture adipose tissue distribution, and emerging evidence suggests that the anatomical location of adipose tissue may be particularly relevant to AF risk. In a meta-analysis of 63 observational studies including more than 350,000 individuals, EAT showed a substantially stronger association with AF than BMI, waist circumference, or waist-to-hip ratio, with particularly strong associations observed for persistent AF [72]. Sex-related differences further modulate this association: although men exhibit a higher absolute incidence of AF, the relative impact of obesity on AF risk may be particularly pronounced in women, potentially reflecting differences in adipose tissue distribution, hormonal milieu, and atrial substrate vulnerability [73]. Obesity also adversely affects AF management, being associated with increased recurrence rates following electrical cardioversion and reduced long-term efficacy of catheter ablation [74].

### 6.2. Ventricular Arrhythmias and Sudden Cardiac Death in Obesity

Clinical studies have shown that obesity is associated with a higher burden of ventricular arrhythmias (VAs) and a greater incidence of appropriate implantable cardioverter-defibrillator (ICD) therapies in high-risk populations.

Large-scale population data have shown that increasing adiposity is associated with QTc prolongation and a higher risk of VAs, especially if metabolic dysfunction is present [75]. In young individuals, obesity is also markedly over-represented among sudden cardiac death (SCD) cases and is associated with a higher prevalence of LV hypertrophy and ventricular fibrillation as the presenting arrest rhythm [76]. However, mechanistic studies remain limited, and the interpretation of these findings requires caution due to confounding factors and limitations inherent in BMI-based classification.

In a MADIT-II subanalysis including non-diabetic patients with prior myocardial infarction, severe left ventricular dysfunction, and an implantable cardioverter-defibrillator (ICD), obesity (BMI ≥ 30 kg/m^2^) was independently associated with a significantly higher risk of sustained VAs. Obese patients had a 64% higher risk of appropriate ICD therapy for ventricular tachycardia/ventricular fibrillation [77]. Furthermore, a subanalysis of the MADIT-CRT trial showed a significant association between BMI and the risk of VAs in white but not black participants [78].

An association between obesity, as well as the severity of obesity, and the risk of SCD has been previously recognized. As early as 1983, a subanalysis of the Framingham Study demonstrated a progressive increase in the incidence of SCD with increasing degrees of obesity, as assessed by Metropolitan Relative Weight (MRW), in both men and women [79]. In a multicenter prospective study involving more than 14,000 men and women with a mean follow-up of 12.6 years, central obesity, as assessed by waist-to-hip ratio (WHR), was independently associated with an increased risk of SCD among non-smokers after adjustment for conventional CV risk factors [80]. Similar findings were reported in a large prospective cohort study of healthy women followed for 32 years, which demonstrated a dose–response relationship between increasing BMI and the risk of SCD (higher risk with BMI ≥ 35 kg/m^2^) [81]. The largest systematic review and dose–response meta-analysis to date, including 14 prospective studies and more than 400,000 participants, confirmed a significant association between adiposity and the risk of SCD. Each 5 kg/m^2^ increase in BMI was associated with a 16% higher risk of SCD, while WHR showed an even stronger association, supporting the importance of central adiposity as a risk marker. Furthermore, the dose–response analysis showed a J-shaped relationship between BMI and SCD, with the lowest risk observed within the normal BMI range and progressively increasing risk across overweight and obese categories [82].

## 7. The Obesity Paradox and Arrhythmias

In cardiac electrophysiology, a distinct dichotomy exists between obesity, arrhythmogenesis, and prognosis, commonly referred to as the obesity paradox. As explained above, excess adiposity directly promotes alterations that increase the incidence of arrhythmias such as AF. Paradoxically, overweight or obese patients with AF show improved survival compared with normal-weight individuals [83]. Researchers speculate that this protective association might be attributed to greater metabolic reserves, higher relative muscle mass, and earlier clinical presentation. Indeed, the concept of the obesity paradox in AF could introduce an important bias in results’ interpretation and must be interpreted with methodological caution. Rather than reflecting a true biological cardioprotection, this phenomenon could be due to these limitations: (1) BMI insufficiency: BMI fails to adequately capture visceral adiposity, and its correlation with EAT remains incompletely characterized, the latter promoting atrial remodeling; (2) cardiorespiratory fitness status: the presence of obese patients with preserved cardiorespiratory fitness (also known as the “fat but fit” phenotype) can lead to improved survival, yet this factor is rarely considered in observational registries, leading to possible misclassification of risk; (3) reverse causation and selection bias: in cohorts with established AF, lower body weight often reflects underlying disease severity or increased frailty, creating a false survival advantage for higher-BMI individuals.

## 8. Obstructive Sleep Apnea Syndrome and Arrhythmogenic Risk

Obesity and OSAS frequently coexist and synergistically contribute to CV disease. The prevalence of OSAS increases with increasing BMI. The likelihood of a higher apnea-hypopnea index (AHI, a measure of OSAS severity) is 14% greater for each one-unit increase in BMI [84]. OSAS-related chronic intermittent hypoxia and intrathoracic pressure swings lead to oxidative stress and inflammation, sympathovagal imbalance, electrical remodeling, and instability. AF represents the most frequent arrhythmia in this setting. Patients with OSAS are at a substantially higher risk of incident AF, with the magnitude of nocturnal oxygen desaturation serving as an independent predictor of future AF [85]. Moreover, patients with untreated OSAS have significantly greater AF recurrence rates after pulmonary vein isolation [86,87].

Beyond AF, individuals with severe sleep-disordered breathing have two- to fourfold higher odds of ventricular ectopy and non-sustained ventricular tachycardia than those without sleep-disordered breathing even after adjustment for potential confounders [88]. Moreover, patients with an ICD and OSAS have a striking increase in the occurrence of VAs during sleeping hours, with no discernible effect on appropriate ICD therapy during nonsleeping hours. These findings further support a potential causal link between the pathophysiology of OSAS and fatal VAs, particularly in patients with a pre-existing arrhythmogenic substrate [89].

OSAS may also promote bradyarrhythmias. The combination of apnea and hypoxemia in patients with OSA elicits an increase in cardiac vagal activation. Sinus pauses, sinus bradycardia, and varying degrees of atrioventricular block are frequently recorded during sleep and are usually temporally linked to apneic events [90,91].

## 9. Reversibility of the Arrhythmogenic Substrate

**Figure 4:**
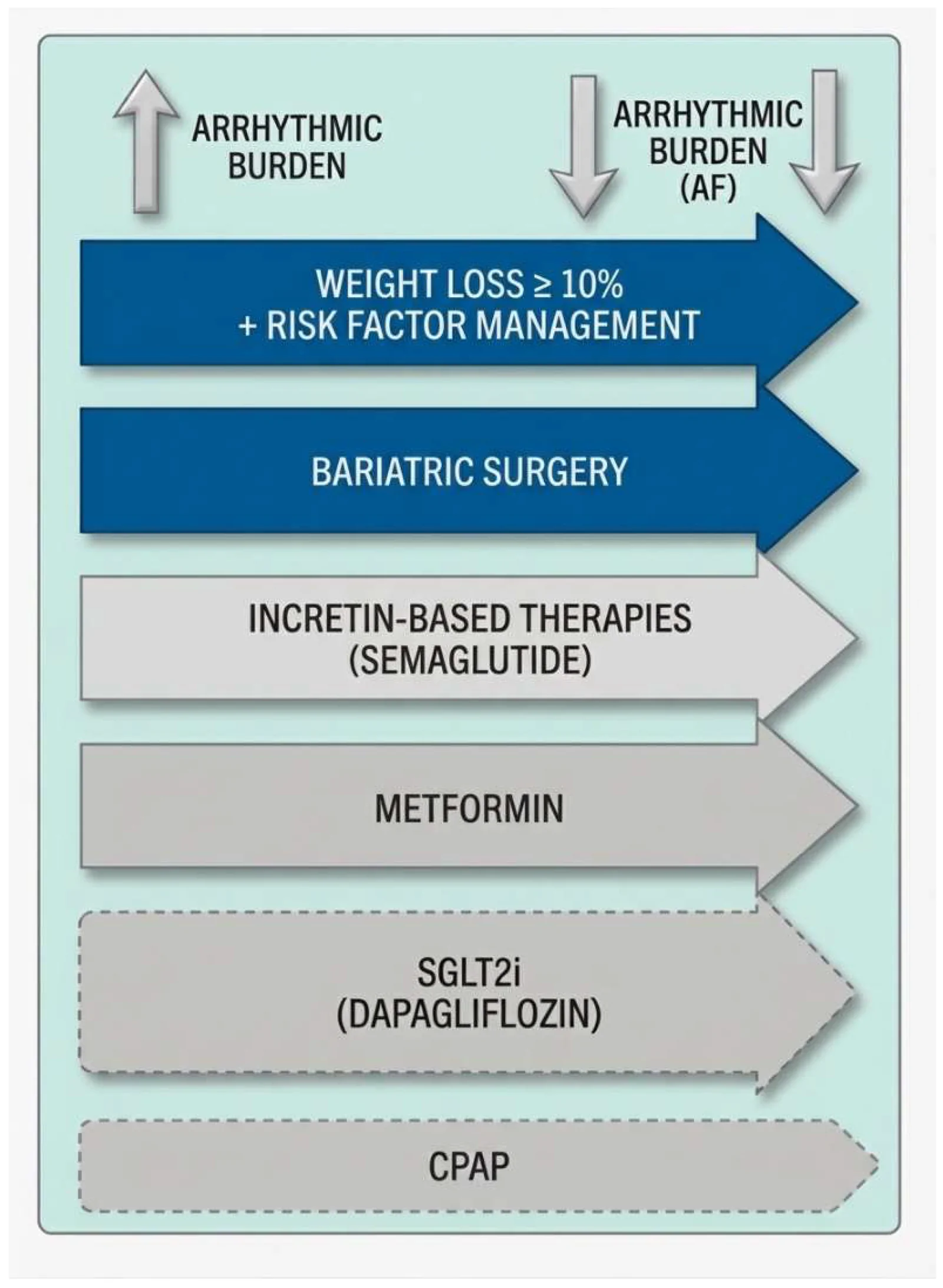
Schematic representation of obesity-related therapeutic measures positively impacting arrhythmic burden. The blue arrows indicate therapies with the strongest clinical evidence, while the grey arrows indicate less strong or inconclusive clinical evidence. The dashed arrows indicate areas with existing, yet limited, clinical evidence. Evidence is mostly focused on atrial fibrillation burden. SGLT2i, Inhibitors of Sodium-Glucose Transporter 2; CPAP, Chronic Positive Airway Pressure. Image created with Gemini AI (3.6 Flash 2026).

Weight-loss strategies encompass a broad spectrum of interventions—ranging from lifestyle modifications to pharmacological therapies and bariatric surgery (BS)—yielding variable degrees of weight reduction. Importantly, in many of the reviewed studies, arrhythmic events were not prespecified primary outcomes; thus, evidence regarding arrhythmogenesis is largely derived from secondary analyses or surrogate markers. Furthermore, patients with obesity frequently receive chronic pharmacological treatments for concomitant comorbidities, which may independently modulate arrhythmic burden. Accordingly, this part focuses on the weight-loss strategies and therapies most commonly used in clinical practice for obese patients, as well as on those for which emerging evidence suggests a potential impact on arrhythmic risk (see Figure 4).

### 9.1. Weight Reduction Strategies

#### 9.1.1. Non-Surgical Weight Loss

Non-surgical weight loss, particularly when achieving ≥10% weight reduction, is associated with substantial improvements in CV risk-factor control and endothelial function [92]. As no studies have specifically addressed the relative contribution of different weight-loss strategies to arrhythmogenesis, in this section we use the term “non-surgical weight loss” broadly to encompass interventions combining dietary modification with lifestyle changes and/or physical exercise. Indeed, most studies use a combination of these measures to achieve weight loss, which alone has not consistently been shown to significantly reduce AF incidence.

In the CARDIO-FIT study, improvement in cardiorespiratory fitness was accompanied by greater reductions in BMI, and the combination of ≥10% weight loss and a ≥2-MET increase in fitness was associated with the greatest freedom from AF, suggesting complementary effects of weight reduction and physical fitness on arrhythmia control [93]. In an RCT including 150 overweight or obese adults with AF, Abed et al. showed that a structured weight-management program combined with intensive risk factor management resulted in significantly greater weight loss and substantial reductions in AF symptom burden and severity, as well as in the number and cumulative duration of AF episodes, compared with general lifestyle advice [94]. In a prospective cohort of overweight/obese patients with AF (LEGACY trial), sustained weight loss (≥10% reduction in body weight) associated with risk factor management was associated with reduction in AF burden, improved symptoms, reduced AF relapse, and favorable reverse remodeling. However, weight fluctuations > 5% attenuated these benefits [95]. In the ARREST-AF trial, comprehensive risk-factor management in obese patients reduced AF burden and duration, improved symptoms, and decreased post-ablation recurrence, alongside significantly greater weight loss compared with controls [96]. Ultimately, the REVERSE-AF study further demonstrated that non-surgical weight loss may favorably modify the natural history of AF, with greater weight loss associated with reduced AF progression and a shift toward less persistent forms [97].

Regarding VAs, current evidence suggests that non-surgical weight loss may improve the ventricular arrhythmogenic substrate by reducing LV hypertrophy, myocardial lipid accumulation, systemic inflammation, and repolarization abnormalities [98]. However, whether these favorable structural and electrophysiological changes translate into a lower incidence of VAs or SCD has yet to be established.

The beneficial effects of non-surgical weight loss on the arrhythmogenic substrate are likely driven by multiple interconnected mechanisms. Indeed, weight loss was shown to change myocardial metabolism and reduce systemic inflammation and EAT depots [99]. It also leads to LA reverse remodeling, with reduced atrial volume and the regression of LA myopathy [94,96,100,101]. LV remodeling after non-surgical weight loss is more challenging to characterize, as findings may vary according to the imaging modality (CMR vs. echocardiography), the surrogate parameter assessed (e.g., LV mass or wall thickness), the inclusion of exercise, and loading conditions. Overall, the available evidence suggests a beneficial effect of non-surgical weight loss on LV mass and wall thickness [96,102,103]. However, studies specifically combining dietary intervention with exercise indicate that changes in CMR-derived LV mass may reflect not only beneficial reverse remodeling related to weight loss but also physiological remodeling induced by exercise. Accordingly, in individuals with obesity undergoing non-surgical weight loss combined with exercise, reductions in LV mass are not consistently observed [104].

Interestingly, Miranda et al. performed single-nucleus RNA sequencing on adipose tissue samples from patients undergoing non-surgical weight loss and showed that weight reduction was associated not only with a reduction in adipose tissue mass but also with a partial reversal of obesity-associated cellular and molecular alterations, including attenuation of inflammatory and senescence-related signatures and restoration of metabolic functions in adipocytes. However, some obesity-associated features, particularly within the immune compartment, persisted after weight loss, suggesting that adipose tissue dysfunction may involve cellular and molecular changes that are not completely reversed by the reduction in tissue mass [4].

#### 9.1.2. Bariatric Surgery

Surgical weight reduction such as BS is consistently associated with a reduced AF burden [105]. Importantly, BS may also modify the arrhythmogenic substrate and improve rhythm-control outcomes in AF. Indeed, BS-induced weight loss was associated with a significant shift toward less advanced AF phenotypes, with a reduction in persistent AF and an increase in paroxysmal AF [106]. In morbidly obese patients undergoing AF ablation, pre-ablation BS was associated with a substantially lower risk of AF recurrence compared with patients without prior BS, with recurrence rates approaching those [107] observed in non-obese individuals [108,109]. However, the available evidence is largely observational and requires further confirmation.

Evidence regarding the effect of BS on VAs is limited. As explained below, small prospective studies have consistently shown favorable changes in ventricular repolarization indices, including QTc and QT dispersion, following BS, suggesting a reduction in ventricular electrical instability. However, these findings are based on electrocardiographic markers rather than clinical arrhythmic events. Interestingly, a recent large propensity-matched real-world study found no significant reduction in incident ventricular tachycardia or ventricular fibrillation after BS [105]. Further research is needed to confirm whether ECG changes translate into a clinically meaningful reduction in VAs after BS.

Atrial remodeling after BS appears to be heterogeneous and time-dependent. Short- to intermediate-term studies have shown improvements in LA size and LA mechanical function, with increases in reservoir and conduit strain [100,110]. However, longer-term data suggest that LA remodeling may not fully reverse: despite sustained improvements in ventricular structure and function, LA volume and estimated filling pressures may increase, while LA reservoir strain may deteriorate [107]. Thus, the literature suggests that after BS, persistent or progressive LA myopathy may remain despite substantial and sustained weight loss. On the other hand, BS-induced weight loss significantly improves LV mass, hypertrophy, and diastolic function, changes observed even in the absence of systemic hypertension [111]. Interestingly, BS-induced positive LV remodeling was observed even in the long term (5.3 years follow-up), with the reductions in LV mass more closely related to decreases in VAT than to changes in BMI [107].

Recent evidence further supports a role for EAT in BS-induced cardiac remodeling. Following laparoscopic sleeve gastrectomy, improvement in LV parameters was accompanied by reduced EAT volume at 12 months, with changes in EAT and several cardiac structural parameters correlating with BMI reduction [112]. In a prospective cohort of BS patients, EAT thickness decreased by 31% after one year, while LV mass and LV mass index decreased and both LV systolic and selected diastolic function parameters improved. Moreover, greater reductions in EAT were independently associated with improvements in global longitudinal strain and septal e′, supporting a potential contribution of EAT reduction to cardiac functional recovery [113].

Strong evidence suggests that BS may also partially reverse the electrophysiological abnormalities found in obesity. In morbidly obese patients, BS was associated with significant reductions in P-wave parameters and LA diameter, suggesting the regression of atrial remodeling and a potential reduction in AF susceptibility [114]. Similarly, improvements in ventricular repolarization indices, including QT dispersion and Tp-e/QT-related parameters, were observed after BS-induced weight loss, possibly leading to a reduced ventricular arrhythmogenic substrate [115,116,117,118].

### 9.2. GLP-1 Agonists and Dual GLP-1/GIP Agonists

The most studied incretin-based therapies include glucagon-like peptide 1 (GLP-1) receptor agonists, such as semaglutide, and dual GIP/GLP-1 receptor agonists, such as tirzepatide. These therapies have demonstrated CV benefits in patients with obesity, including reductions in major adverse CV events. However, whether these benefits are independent of weight loss remains uncertain. Evidence regarding arrhythmogenesis is still limited, focusing predominantly on AF, and is largely derived from studies or meta-analyses that do not specifically stratify outcomes according to BMI, visceral adiposity, or diabetes.

Available evidence suggests an anti-arrhythmogenic role of incretin-based therapies for AF. A meta-analysis of RCTs reported a 42% reduction in incident AF among patients (mostly diabetic) receiving semaglutide versus placebo [119]. A 2024 meta-analysis of 16 RCTs including 19,892 patients found that semaglutide was associated with a significantly lower risk of new-onset AF, irrespective of diabetic status. No significant differences were observed for VAs or SCD. Ultimately, in a recent meta-analysis of 24 RCTs and >40,000 participants with overweight or obesity, incretin-based therapies were associated with an 18% relative reduction in AF risk compared with placebo, which was not affected by the magnitude of weight loss [120].

While GLP-1 agonists have been associated with a lower risk of AF, studies evaluating tirzepatide alone have not demonstrated a significant AF reduction compared with placebo, despite the evidence of favorable cardiac remodeling in patients with obesity-related HFpEF treated with tirzepatide [121].

Available evidence has not demonstrated a significant effect of incretin-based therapies on VAs or SCD. Meta-analyses of randomized trials on overweight/obese patients (diabetic and non-diabetic) have shown no significant reduction in VAs or ventricular fibrillation with semaglutide, although limited by the relatively low number of events [122]. Overall, incretin-based therapies (especially semaglutide) appear to have a neutral rather than antiarrhythmic effect on VAs, with no consistent evidence of increased ventricular proarrhythmic risk [123].

Although not primarily designed to assess LA structure, trials with semaglutide have demonstrated improved filling pressures and improved cardiac function, changes expected to reduce chronic LA pressure and volume overload, major determinants of LA remodeling. Recently, studies have focused more on the role of GLP-1-based therapies on cardiac remodeling in obesity-related HFpEF. Semaglutide was shown to attenuate LA remodeling and RV enlargement and improve indices of LV diastolic function, although no significant changes in LV mass, dimensions, or systolic function were observed [124]. Tirzepatide, in contrast, was associated with a significant reduction in LV mass and paracardiac adipose tissue on CMR [121]. This evidence suggests that these therapies may exert beneficial, potentially disease-modifying effects on obesity-related cardiac remodeling, although the relative contribution of weight loss itself versus weight-independent metabolic and hemodynamic mechanisms remains uncertain.

Preclinical studies suggest that GLP-1 agonism can reduce atrial fibrosis and adverse atrial structural remodeling, thereby decreasing AF susceptibility [125]. In a translational study, Basdas et al. showed that semaglutide modulates pro-inflammatory adipogenesis in human EAT, reducing the expression of inflammatory markers such as FABP4 and sPLA2. Moreover, conditioned media from semaglutide-treated EAT altered paracrine signaling to human atrial cardiomyocytes, suggesting that modulation of EAT inflammation may influence the atrial arrhythmogenic substrate [126]. These findings provide a potential mechanistic link between GLP-1 receptor activation, epicardial fat remodeling, and atrial arrhythmogenesis, although their clinical relevance remains to be determined.

The partially conflicting data regarding incretin-based therapies compared to BS may be partially explained by the systemic impact of BS, which, parallel to weight loss, can positively control CV risk factors such as systemic hypertension, diabetes, and dyslipidemia. These systemic metabolic shifts might influence arrhythmogenic mechanisms, which may account for the current, less conclusive data observed with incretin-based therapies.

### 9.3. OSAS Management

Given the close relationship between obesity, OSAS, and arrhythmogenic risk, sustained weight loss represents a key upstream therapeutic strategy. Weight loss ≥ 10% resulted in a 6-fold greater probability of maintenance of sinus rhythm in a cohort of 1415 consecutive patients with AF and BMI ≥ 27 kg/m^2^ [95]. While observational data suggest a potential benefit of chronic positive airway pressure (CPAP) in reducing AF recurrence, randomized trials have not confirmed this effect [91,127]. Similarly, evidence regarding the efficacy of CPAP in treating OSAS-related bradyarrhythmias remains limited and inconsistent [128]. In conclusion, while promising, the available evidence remains limited, and further studies are needed to establish its efficacy.

### 9.4. Inhibitors of Sodium Glucose Transporter 2

Although most studies investigating the effects of SGLT2 inhibitors (SGLT2i) on arrhythmogenesis have been conducted in patients with type 2 diabetes rather than in individuals with obesity per se, available evidence suggests a potential favorable effect on arrhythmic risk, particularly for AF. In the DECLARE-TIMI 58 trial, dapagliflozin reduced AF/atrial flutter events with a consistent relative benefit across BMI categories and a potentially greater absolute benefit among obese patients (BMI ≥ 30 kg/m^2^) [129]. Meta-analyses of randomized trials have subsequently confirmed a modest reduction in AF/atrial arrhythmias, whereas no significant effect on VAs has been demonstrated [130]. An observational study on >10,000 overweight diabetic subjects found that ≥5% weight loss during treatment was associated with a significant reduction in new-onset AF [131]. However, given the small amount of evidence on obesity and arrhythmic risk in patients treated with SGLT2i, these data remain preliminary and will require further mechanistic confirmation. Also, whether these effects are mediated primarily by weight loss, improved hemodynamic loading conditions, metabolic and inflammatory effects, or favorable atrial remodeling remains uncertain.

### 9.5. Metformin

Diabetes induces multiple metabolic and molecular alterations that may promote arrhythmogenesis. Glycemic variability, oxidative stress, and chronic systemic inflammation can contribute to electrical, structural, and autonomic remodeling, particularly affecting the LA. Although metformin induces little or no clinically meaningful weight loss, it remains widely used in patients with obesity and type 2 diabetes and exerts effects that extend beyond glycemic control. Experimental studies have suggested protective effects against both atrial and ventricular arrhythmias, potentially mediated by AMPK activation, improved calcium handling, reduced oxidative stress, and preservation of connexin-43 signaling [132]. Clinical evidence suggests that metformin use in patients with type 2 diabetes is associated with a reduced risk of incident AF. However, these studies were not specifically conducted in obese populations, and BMI was either not reported or was generally within the normal range [133,134]. Interestingly, preliminary results from the recent META-AF trial suggest that at 12 months from AF ablation, freedom from recurrent arrhythmias is higher in overweight, non-diabetic patients treated with metformin, with minimal changes in weight.

Evidence regarding the association between metformin use and VAs remains limited, particularly in patients with obesity. Some observational studies have reported lower rates of VAs, including ventricular tachycardia and ventricular fibrillation, among patients treated with metformin compared with those receiving sulfonylureas [135]. However, these findings require further confirmation and are not consistently derived from studies specifically conducted in obese populations.

These data on metformin and arrhythmic risk in overweight or obese patients are particularly intriguing; given the modest weight loss achieved with this treatment, they point to potential weight-independent mechanisms in arrhythmogenesis. Nevertheless, dedicated studies are needed to dissect the specific metabolic, inflammatory, and weight-related changes across distinct BMI categories, thereby confirming the therapeutic role of metformin in improving arrhythmic outcomes.

### 9.6. Bupropione/Naltrexone and Orlistat

Bupropion/naltrexone is a centrally acting anti-obesity therapy that promotes weight loss through modulation of hypothalamic appetite regulation and reward-related pathways. Orlistat is a peripherally acting lipase inhibitor that induces weight loss by reducing intestinal fat absorption and caloric intake. Unlike GLP-1 receptor agonists and bariatric surgery, there is currently no evidence that bupropion reduces arrhythmogenic risk. Given its sympathomimetic properties and potential to increase heart rate and blood pressure, bupropion is not generally considered a therapy capable of favorably modifying the arrhythmogenic substrate associated with obesity. Regarding orlistat, evidence supporting a direct antiarrhythmic effect is currently lacking. Although modest weight loss and improvements in cardiometabolic risk factors may theoretically attenuate obesity-related arrhythmogenic mechanisms, no studies have demonstrated a reduction in atrial or ventricular arrhythmias secondary to orlistat therapy.

### 9.7. Epicardial EVs: A Possible Anti-Arrhythmic Target

Given the central role of obese EAT in driving atrial remodeling, therapeutic strategies aiming to modulate EAT inflammation, oxidative stress, and EV secretion are actively being explored. Validated therapeutic strategies specifically targeting the EAT secretome are currently lacking. However, recent bioengineering approaches have utilized engineered vesicle systems for targeted therapeutic delivery to EAT. Wang et al. synthesized adipose-derived mesenchymal stem cell (ADSC) exosome-liposome hybrid nanoparticles loaded with a GLP-1R/GIPR dual-target agonist. This nanotherapeutic approach specifically targets EAT in obese animal models, suppressing ferroptosis by restoring glutathione (GSH) levels and GPX4 expression while dramatically reducing ROS, lipid peroxidation markers, and inflammatory cytokines (IL-1β, IL-6, MCP-1) [136]. While these preliminary observations suggest that targeting the EAT microenvironment could theoretically reduce atrial fibrosis and lower susceptibility to pacing-induced AF, significant challenges remain regarding the scalable manufacturing, long-term safety, and clinical translation of such complex hybrid nanotherapeutics. In addition, definitive methods to verify the adipocyte-specific origin of these EVs must be established, alongside comprehensive profiling of their proteomic cargo. Most importantly, targeted clinical trials will be necessary to validate the translational relevance of these preliminary preclinical insights.

## 10. Long-Term Maintenance Strategies

Few studies report long-term outcomes (>5 years) of different therapeutic strategies on arrhythmic burden in obesity, and they are mostly focused on non-surgical weight loss and bariatric surgery. Evidence from the LEGACY trial (median follow-up period of 5 years) underlined how patients experiencing weight fluctuations over the observation time had a 2-fold higher risk of AF recurrence, suggesting that linear, sustained weight loss is clinically superior in maintaining sinus rhythm [95]. Furthermore, large registries have shown that BS is associated with a significant reduction in new-onset AF incidence over a median follow-up of 19 years [137]. While these encouraging data highlight the critical role of weight-dependent mechanisms in arrhythmogenesis, further long-term research is warranted to validate the sustained efficacy of diverse therapeutic interventions on arrhythmia control in obese populations.

## 11. Conclusions and Future Perspectives

Given its increasing global prevalence, obesity has emerged as a major and growing public health concern. Through systemic mechanisms including VAT dysfunction, paracrine pathways related to EAT remodeling, associated comorbidities, and mechanical alterations such as increased volume and pressure overload on the myocardium, obesity represents a major challenge for the prevention and management of cardiac arrhythmias, particularly AF.

The current body of literature relies heavily on observational human data and preclinical models, offering primarily indirect and correlational evidence rather than definitive proof of causality. The limited number of studies specifically addressing the interplay between obesity and arrhythmogenesis—particularly the mechanisms underlying arrhythmic risk in otherwise healthy individuals with obesity—makes it difficult to identify the key pro-arrhythmic pathways operating in this population and, consequently, to target them effectively. Data reviewed here highlight the potential role of dysfunctional adipose tissue, involving both VAT and EAT. In obesity, these depots undergo profound structural and functional remodeling, leading to systemic and paracrine release of mediators that promote local inflammation, fibrosis, structural cardiac remodeling, and electrical instability. Among these mediators, EVs emerge as a potentially important intercellular signaling mechanism linking dysfunctional adipose tissue to cardiac remodeling and arrhythmogenesis.

Human genetic evidence, particularly Mendelian randomization studies using large-scale cohorts, provides mechanistic insights by supporting a causal link between genetically predicted overall adiposity or visceral fat and an increased risk of AF. Conversely, genetic data regarding specific circulating adipokines (adiponectin, leptin) often fail to consistently demonstrate direct causality, as findings are frequently limited by a lack of consistent replication across large cohorts, as well as confounding effects from underlying metabolic dysfunction, including insulin resistance and systemic hypertension [138].

Furthermore, the lack of clinical trials specifically enrolling patients with obesity—particularly those with otherwise uncomplicated obesity—and the absence of a standardized measure of visceral adiposity for patient stratification severely limit our ability to establish direct causality or isolate EAT/VAT-specific effects from general systemic obesity. This limitation is particularly relevant when evaluating pharmacological therapies, as it remains unclear whether observed anti-arrhythmic effects are primarily mediated by weight loss per se or instead reflect direct, weight-independent metabolic, inflammatory, or electrophysiological mechanisms.

Further research—especially prospective interventional trials and studies directly linking specific adipo-secretomic profiles to clinical arrhythmic outcomes—is therefore warranted to disentangle the intrinsic, causal effects of obesity from those attributable to its associated comorbidities and to elucidate the mechanisms underlying the clinical and structural improvements that ultimately reduce arrhythmogenic potential.

## 12. Limitations

Despite the growing body of evidence linking adipose tissue remodeling to a potentially pro-arrhythmic cardiac substrate, the mechanisms underlying this association remain incompletely understood. The limitations of this narrative review are represented, unlike for a systematic review, by the presence of a possible selection bias as well as quality subjectivity. Several limitations of the available literature should also be considered.

First, studies often analyze overweight and obesity as relatively broad categories, with limited stratification according to the severity of obesity and the distribution of adipose tissue. Moreover, BMI does not capture differences in body composition or fat distribution, particularly the accumulation of VAT, which appears to be more closely associated with adverse cardiometabolic and CV outcomes. These limitations may hinder the identification of dose-dependent relationships between the extent and distribution of adipose excess and the development of structural or electrical abnormalities potentially contributing to arrhythmogenesis.

Second, a substantial proportion of the available evidence derives from preclinical models, limiting the extent to which these findings can be directly extrapolated to human pathophysiology.

Third, much of the clinical evidence remains indirect, demonstrating associations between obesity and structural or electrophysiological cardiac alterations without establishing whether, or to what extent, these changes translate into a higher incidence of clinically manifest arrhythmias.

Fourth, it remains unclear whether and how EAT and VAT remodeling evolve in parallel with advancing degrees of obesity, or whether—following the initial trigger of caloric excess—these depots eventually become metabolically autonomous, sustaining a pro-arrhythmic stimulus even after weight loss.

Finally, evidence regarding the reversibility of the potential arrhythmic substrate remains limited, and the mechanisms underlying such reversibility are still poorly defined. In particular, it remains unclear whether regression of the arrhythmic substrate is primarily related to the extent of weight reduction itself or to the reversal of the metabolic, inflammatory, and hemodynamic abnormalities accompanying obesity. These limitations highlight the need for longitudinal, mechanistic studies integrating the degree, distribution, and duration of adipose excess with detailed structural, electrical, and metabolic phenotyping, both before and after weight reduction.

## Data Availability

No new data were created or analyzed in this study. Data sharing is not applicable to this article.

## Supplementary Materials

The following supporting information can be downloaded at: https://www.mdpi.com/article/10.3390/biomedicines14102269/s1, Table S1: The table summarizes the most recent translational and clinical evidence focused on overweight/obesity and VAT secretion, divided in clinical studies and other (mixed studies); Table S2: The table summarizes the most recent translational and clinical evidence focused on overweight/obesity and EAT paracrine function, divided in clinical studies and other (mixed studies).
