# Supplementary material for: Obesity, Metabolic Modulation, and Arrhythmias: From Adipose Tissue Dysfunction to a Modifiable Arrhythmogenic Substrate
Source: Biomedicines. 2026 Oct 7;14(10):2269. doi: 10.3390/biomedicines14102269 (PMC13701308; doi:10.3390/biomedicines14102269)
Supplement: Supplementary file 1 [file biomedicines-14-02269-s001.zip › biomedicines-4567254-supplementary.pdf]

Supplementary Table S1: The table summarizes the most recent translational and clinical evidence focused on overweight/obesity and VAT secretion, divided in clinical studies and other (mixed studies). 11β-HSD: 11β-Hydroxysteroid dehydrogenase, 4-HNE: 4-Hydroxynonenal, AT: adipose tissue, ATM: Adipose Tissue Macrophage, BMI: body mass index, BrAT: breast adipose tissue, CCND1: cyclin D1, CD14: Cluster of Differentiation 14, CD45: Cluster of Differentiation 45, CD68: Cluster of Differentiation 68, CRP: C-reactive protein, CT: computed tomography, CVD: cardiovascular diseases, DM: diabetes mellitus, DPP4: Dipeptidyl Peptidase-4, ELISA: enzyme-linked immunosorbent assay, FABP4: Fatty Acid Binding Protein 4, FFA: free fatty acids, GAT: Gluteal adipose tissue, IL-18: interleukin 18, IL-1β: interleukin 1 beta, IL-6: interleukin 6, IL-8: interleukin 8, LAGB: laparoscopic adjustable gastric band, MCP-1: monocyte chemoattractant protein 1, NAFL: Non-Alcoholic Fatty Liver, NAFLD: Non-Alcoholic Fatty Liver Disease, NASH: Non-Alcoholic Steatohepatitis, NLRP3: Nucleotide-binding Oligomerization Domain-like Receptor Protein 3, NPY: neuropeptide Y, PPARGC1B: PPAR gamma coactivator 1-beta, RT-qPCR: real time quantitative polymerase chain reaction, SAT: subcutaneous adipose tissue, SVCs: stromal vascular cells, SVF: stromal vascular fraction, THE: tetrahydrocortisone, THF: tetrahydrocortisol, TNF-α: tumor necrosis factor alpha, TSC22D3: TSC22 Domain Family Member 3, TZD: Thiazolidinediones, VAT: visceral adipose tissue, WAT: white adipose tissue.

Reference	Population	BMI (Kg/m2)	VAT characteristics	Methods	Conclusions
**Clinical studies**					
Soren Nielsen et al. (2004)<br><br>DOI: 10.1172/JCI21047	Obese subjects, n=44<br>Healthy controls, n=24<br><br>Obese male (OM), n=20<br>Obese female (OF), n=24<br>Lean male (LM), n=12<br>Lean female (LF), n=12	LF 21.6 ± 0.7<br><br>OF 33.8 ± 0.5<br><br>LM 23.6 ± 0.5<br><br>OM 32.9 ± 0.7	VAT area measurement (CT-scan)<br><br>LF 17 ± 3 cm^2<br>OF 130 ± 10 cm^2<br>LM 72 ± 13 cm^2<br>OM 232 ± 15 cm^2	• FFA measurement (isotope dilution technique) of systemic, leg, splanchnic delivery on hepatic arterial/venous blood samples.	VAT: ↑ FFA production and delivery to the liver with ↑ VAT (greater effect in women vs men).<br>VAT pathogenic role in metabolic syndrome via FFA production (lipolysis)?

Jens M. Bruun et al. (2005)<br><br>DOI: 10.1210/jc.2004-1696	Patients undergoing surgery, n=10<br><br>Healthy controls, n=5<br><br>Obese subjects, n=5	Control 25.1 ± 1.3<br><br>Obese 43 ± 3.3	N/A	• Intraoperative adipose tissue (SAT/VAT) biopsy collection;<br>• Cultured EAT/SAT samples with cytokines/antidiabetic componds and conditioned media collection (15-72h);<br>• AT-derived SVCs and adipocytes isolation from SAT/VAT samples;<br>• Protein (MCP-1) expression analysis (ELISA) on SAT/VAT-conditioned media;<br>• Gene (MCP-1, TNF-α, IL-8, IL-6, leptin, CD68, CD14) expression analysis (RT-qPCR) on SAT/VAT and AT-derived cells	VAT: ↑ macrophage infiltration and pro-inflammatory adipokines. ↑ MCP-1 production by SVCs (macrophages) vs adipocytes. ↑ MCP-1 release in obese subjects (vs SAT/healthy controls) controls.<br>↑ inflammation → ↑ MCP-1 production and release.<br>Anti-diabetics drugs (metformin/TZD) → ↓ MCP-1 production and release in VAT/SAT→anti-inflammatory effect?
Stephanie Barth et al (2010)<br><br>DOI: 10.1159/000319508	(A) Surgical obese patients (LAGB), n=31<br><br>(B) Surgical controls, n=31<br><br>(C) Non-surgical obese patients, n=76<br><br>(D) Non-surgical controls, n=30	A 48.2 ± 6.4<br>B 27.7 ± 3.5<br>C 49.1 ± 8.8<br>D 26.7 ± 4.1	N/A	• Preoperative peripheral blood and urine sample collection;<br>• Intraoperative adipose tissue (SAT/VAT) biopsy collection (A+B, n=62);<br>• NPY measurement (radioimmunoassay) on plasma samples;<br>• Cortisol measurement (electrochemiluminescence immunoassay) on serum samples;<br>• Cortisol metabolites measurment (gas chromatography/mass spectrometry) on urine samples -->(allo-THF+THF)/THE ratio as an index of 11β-HSD activity<br>• Gene (NPY, omentin, visfatin) expression analysis (RT-qPCR)	Circulation: NPY levels and glucocorticoid measures not elevated In the obese patients.<br>VAT/SAT: NPY, omentin, and visfatin expression comparable between obese individuals and controls.<br>Consideration on total production rate adjusted for adipose mass?

H. N. Heneghan et al. (2011) DOI: 10.1210/jc.2010-2701	Obese subjects, n=52<br><br>Leans subjects, n=32	Obese >40<br><br>Lean <25	N/A	• Preoperative peripheral blood sample collection (obese n=30, lean n=20);<br>• Intraoperative adipose tissue (SAT/VAT) biopsy collection (obese n=22, lean n=12);<br>• miRNA discovery expression (microarray) and pathway analysis on adipose tissue (SAT/VAT) samples (obese n=3, lean n=2);<br>• Validation of candidate miRNAs in EAT/SAT samples (RT-qPCR, obese n=19, lean n=10);<br>• Validation of candidate miRNAs in blood samples (RT-qPCR, obese n=30, lean n=32);	VAT and circulation: ↓ miR-17–5p and miR-132, inverse correlation miR-17-5p and BMI in obese patients.<br>miRNA profile as non-invasive biomarkers of obesity-related alterations?
Ximena Terra et al. (2012) DOI: 10.1111/j.1365-2265.2011.04327.x	Female surgery patients, n=133<br><br>Control patients, n=40<br><br>Obese patients, n=93<br><br>Follow-up obese cohort, n=30	Control 21.9 ± 0.3<br><br>Obese 47.9 ± 0.7<br>Obese -DM 48.8 ± 1.2<br>Obese no-DM 47.8 ± 0.8	N/A	• Preoperative peripheral blood sample collection<br>• Intraoperative adipose tissue (SAT/VAT) biopsy collection (abdominal surgery);<br>• Gene (visfatin, adiponectin, resistin, TNF-α, IL-6) expression (RT-PCR) (obese n=50, control n=8)<br>• Protein (visfatin, adiponectin, IL-6, CRP, resistin, and TNF-α) expression analysis (ELISA) on blood samples;<br>• Protein (visfatin) expression analysis (ELISA) on blood samples (follow up at 6-12 months);	VAT/SAT and circulation: ↑ visfatin in obese patients (independent of DM status). Positive association with pro-inflammatory markers (IL-6, TNF-α, CRP) and negative association with adiponectin.<br>Visfatin role as a pro-inflammatory adipocytokine and not insulin-mimetic/glucose-regulatory in severe obesity?

Nathalie Esser et al. (2013)<br><br>DOI: 10.1007/s00125-013-3023-9	Bariatric surgery patients, n=44<br><br>Metabolically healthy obese (MHO), n=21<br><br>Metabolically unhealthy obese (MUO), n=23<br><br>Lean patients, n= 9	Lean 22.7 ± 0.9<br><br>(MHO) 42.3 ± 1<br><br>[MUO] 42.7 ± 1.1	N/A	• Intraoperative adipose tissue (SAT/VAT) biopsy collection;<br>• Cultured EAT/SAT samples and conditioned media collection (22h);<br>• Gene (IL1B, IL18, inflammasome genes) expression analysis (RT-qPCR) on SAT/VAT samples;<br>• AT-derived SVCs and ATMs isolation (SAT/VAT samples) and characterization (flow cytometry);<br>• Protein (IL-1β and IL-18) expression analysis (ELISA) on isolated ATMs and SAT/VAT and conditioned media samples;<br>• Caspase-1 activity assay (flow cytometry) on AT-derived SVCs.	VAT: ↑ proinflammatory-macrophages (ATMs, granulocytes) infiltration and ↓ anti-inflammatory regulatory T cells in MUO patients → ↑ inflammasome activation, caspase-1 activity and IL-1β, IL-18, NLRP3 secretion.
Henrik Svensson et al. (2014)<br><br>DOI: 10.1186/1472-6823-14-7	Abdominal surgery patients, n=17	35.8 ± 10.8	N/A	• Intraoperative adipose tissue (SAT, n=17/VAT, n=11) biopsy collection;<br>• Cultured SAT/VAT samples and cultured media collection (at 2, 4, 6, 8, 24 h);<br>• Cytokine assay (multiplex immunoassay, ELISA) on cultured media samples;<br>• Immunohistochemistry on SAT/VAT samples;	VAT: depot-specific production and secretion of adipokines (chemerin, cytokines (except IL-6), and DPP4) and macrophages infiltration<br>More inflammation-prone tissue vs SAT?
Antônio Sérgio Barcala Jorge et al. (2016)<br><br>DOI: 10.1	Bariatric surgery patients, n=21<br><br>NAFL patients, n=8<br>NASH patients, n=13	NAFL 40.1 ± 1.5<br><br>NASH 44.1 ± 4.5	N/A	• Preoperative peripheral blood sample collection (serum);<br>• Intraoperative WAT and liver biopsy collection;<br>• Immunohistochemistry on liver biopsies;<br>• Gene (IL-6, TNF-α) expression analysis (RT-qPCR) on WAT	WAT: ↑ IL-6/TNF-α expression and BMI positively correlated to NAFLD severity. Feedback loop with inflammation and metabolic disturbances (dyslipidemia/IR) and NAFLD progression in obese patients?

016/j.orcp.2016.03.009				and liver samples	
Morana Jaganjac et al. (2017)<br><br>DOI: 10.1016/j.redox.2017.03.012	Weight reduction surgery patients, n=40<br><br>Insulin-sensitive (IS), n=8<br>Insulin resistance (IR), n=21<br>DM, n=11	IS 43.2 ± 4.7<br><br>IR 44.3 ± 7.5<br><br>DM 42.9 ± 6.9	N/A	• Preoperative peripheral blood sample collection;<br>• Intraoperative VAT biopsy collection;<br>• Immunohistochemistry (4-HNE) on VAT samples;<br>• VAT-derived SVF cells and adipocyte isolation;<br>• In vitro conditioning (4-HNE, metformin, metformin+insulin) of VAT-adipocytes;<br>• Gene (CCND1, FABP4, TSC22D3 e PPARGC1B) expression analysis (RT-qPCR) on VAT-adipocytes;	VAT: smaller adipocytes, ↑ macrophagic infiltration, and ↑ 4-HNE buildup (also in circulation) in IR/DM patients. ↓ macrophagic infiltration and ↓ 4-HNE buildup in metformin+insulin treated patients.<br>Pathogenic/biomarker role of 4-HNE for VAT dysfunction?
Aleksandra Korac et al. (2021)<br><br>DOI: 10.5114/aoms/92118	Elective surgery patients, n=50<br><br>Obese patients, n=30<br><br>Normal weight patients, n= 20	N/A	N/A	• Preoperative peripheral blood sample collection (plasma/serum);<br>• Intraoperative adipose tissue (SAT/VAT) biopsy collection;<br>• Gene (leptin, adiponectin, and resistin) expression analysis (RT-qPCR) on SAT/VAT samples;<br>• Protein (leptin, adiponectin, and resistin) expression analysis (western blot) on plasma/serum and SAT/VAT samples;<br>• Immunohistoche	VAT/SAT: ↑ resistin, ↓adiponectin and discordant leptin levels in obese patients<br>Endocrine dysfunction as an early sign of metabolic syndrome onset?

				mistry (leptin) on SAT/VAT samples;	
**Evidence in human and animal model**					
Philip G McTernan (2002)<br><br>DOI: 10.1210/jcem.87.5.8627	Elective surgery patients, n=45	26 ± 3.8	N/A	• Intraoperative adipose tissue (SAT, n=19/VAT, n=10/GAT n=9/BrAT, n=7) biopsy collection;<br>• Pre-adipocytes and adipocytes isolation from SAT/VAT samples and conditioned media collection;<br>• Gene (resistin, CD45) expression analysis (RT-qPCR) on AT-derived cells;<br>• Protein (resistin/resistin-like protein) expression analysis (western blot, ELISA) on AT-derived cells conditioned media;<br>• Immunohistochemistry (resistin, CD45) on SAT/Rat AT samples	↑ resistin expression (mRNA/protein) SAT/VAT vs GAT/BrAT and predominantly originating from pre-adipocytes rather than mature adipocytes.<br>Role for resistin as link in central obesity-T2D-CVD?

Supplementary Table S2: The table summarizes the most recent translational and clinical evidence focused on overweight/obesity and EAT paracrine function, divided in clinical studies and other (mixed studies). ACE: angiotensin-converting enzyme, AF: atrial fibrillation, AGT: angiotensinogen, AGTR1: angiotensin II receptor type 1, AT: adipose tissue, BSA: body surface area, CABG: coronary artery bypass graft, CAD: coronary artery disease, CCM: cardiomyocytes conditioned medium, CD45: Cluster of Differentiation 45, CHD: coronary artery disease, COL1A1: Collagen type I alpha 1, CPR: C-reactive protein, CT: computed tomography, DM: diabetes mellitus, EAT: epicardial adipose tissue, ECM: extracellular matrix, ELISA: enzyme-linked immunosorbent assay, EVs: extracellular vescicles, FN1: Fibronectin 1, GAT: gluteal Adipose Tissue, H9c2: rat myoblast cell line, HF: heart failure, Hi-iLAEAT: high iLAEAT, HTN: systemic hypertension, HUVEC: Human umbilical vein endothelial cells, IFN-γ: interferon gamma, IL- 1ra: Interleukin-1 receptor antagonist, IL-18: interleukin 18, IL-1β: interleukin 1 beta, IL-1β: interleukin 1 beta, IL-6: interleukin 6, IL-6sR: IL-6 receptor, iLAEAT: indexed left atrial epicardial adipose tissue, Int-iLAEAT: intermediate iLAEAT, iPSC-CM: induced pluripotent stem cells cardiomyocytes, Kcjn12: Potassium Inwardly Rectifying Channel Subfamily J Member 12, Kcnj2: Potassium Inwardly Rectifying Channel Subfamily J Member 2, LA: left atrium, LAA: left atrial appendage, LC-MS: liquid chromatography–mass spectrometry, Low-iLAEAT: low iLAEAT, LV: left ventricle, MCP-1: monocyte chemoattractant protein 1, MCS: mesenchymal stromal cell, MMP8: Matrix metalloproteinase-8, MPO: Myeloperoxidase, MRI: Magnetic resonance imaging, NADPH: Nicotinamide adenine dinucleotide phosphate, NET: Neutrophil Extracellular Trap,

NHCF: normal heart atrial cardiac fibroblasts, NLRP3: Nucleotide-binding Oligomerization Domain-like Receptor Protein 3, NRVMs: neonatal rat ventricular myocytes, NTA: nanoparticle tracking analysis, OAT: omental adipose tissue, PAI -1: Plasminogen Activator Inhibitor-1, POAF: post-operative atrial fibrillation, PVAT perivascular adipose tissue, RAA: right atrial appendage, Rac1: Ras-related C3 botulinum toxin substrate 1, RT-qPCR: real time quantitative polymerase chain reaction, RV: right ventricle, SAT: subcutaneous adipose tissue, SCD: sudden cardiac death, SFRP5: Secreted Frizzled Related Protein 5, TEM; transmission electron microscopy, TGF β1: Transforming Growth Factor Beta 1, TNF-α: tumor necrosis factor alpha, t-PA: Tissue-type Plasminogen Activator, VR: valvular replacement, WNT5A: Wnt Family Member 5A.

Reference	Population	BMI (Kg/m2)	EAT characteristics	Methods	Conclusions
**Clinical studies - miRNA**					
Yang Liu et al. (2016) DOI: 10.1155/2016/1629236	CAD+DM patients, n=5<br><br>No-CAD+DM patients, n=5	CAD+DM 28.6 ± 4<br><br>No-CAD+DM 29.5 ± 6	N/A	• Intraoperative EAT sample collection;<br>• miRNA profiling (whole genome miRNA expression microarrays) and bioinformatic analysis on EAT samples	EAT: dysregulated miRNA profile (CAD-DM). Role in CAD and/or type 2 DM pathogenesis ? miR-4687-3p role in insulin signaling pathway?
Khanh-van Tran et al. (2019) DOI: 10.3389/fcvm.2019.00115	AF-patients, n=91<br><br>Low-iLAEAT, n=30<br><br>Int-iLAEAT, n=31<br><br>Hi-iLAEAT, n=30	Low-iLAEAT 31 ± 7<br><br>Int-iLAEAT 32 ± 6<br><br>Hi-iLAEAT 32 ± 5	EAT surrounding LA measurement (CT scan, echocardiogram) indexed to BSA→ iLAEAT	• Preoperative venous blood sample collection (plasma)<br>• miRNAs (86 commonly expressed in cardiomyocyte) profiling (RT-qPCR) and pathway analysis on plasma samples (n=91)	Plasma: miRNAs 155-5p/302a-3p positive association with Hi-iLAEAT. Role in development and maintenance of AF (via inflammation, adipogenesis and adverse cardiac structural remodeling)?

Josep Marí-Alexandre et al. (2019)<br><br>DOI: 10.1016/j.rec.2017.12.007	CHD-SCD, n=155<br><br>no-CHD-SCD, n=84	CHD-SCD 28.7 ± 5.4<br><br>no-CHD-SD 26.5 ± 4.7	EAT thickness measurement (autoptic examination) → EAT score (total amount, mm).<br><br>CHD-SCD (n=130) 53 ± 18.2 mm<br><br>no-CHD-SCD (n=73) 39.6 ± 16.1 mm	• Autoptic EAT and liver biopsy collection;<br>• miRNA profiling (expression microarrays) on EAT samples (CHD-SCD, n=3; no-CHD-SD, n=3);<br>• Validation (qRT-PCR) of candidate miRNAs in:<br>– EAT samples (CHD-SCD, n=99; no-CHD-SD, n=28)<br>– liver samples (CHD-SCD, n=62; no-CHD-SD, n=23)	EAT: hypertrophic and dysfunctional (CHD-SCD). EAT/liver: miR-34a-5p ↑ and positive correlation in CRP in stenotic-CHD-SCD vs no-stenotic-CHD-SD/ no-CHD-SD. Early/systemic role of miR-34a-5p in CHD onset?
Joanna Szydełko et al. (2026)<br><br>DOI: 10.1186/s12933-025-03055-2	Type 2 DM-CAD patients, n = 30<br><br>Type 2 DM patients, n = 30<br><br>CAD patients, n = 16<br><br>Healthy controls, n=18	Type 2 DM-CAD 29.6 ± 4<br><br>Type 2 DM 31.1 ± 3<br><br>CAD 28.6 ± 3<br><br>Controls 29.7 ± 4	EAT thickness measurement (echocardiography)<br><br>Type 2 DM-CAD 7.78 (6.97–9.41) mm<br><br>Type 2 DM 5.46 (5.05–6.76) mm<br><br>CAD 6.91 (5.41–8.05) mm<br><br>Controls 4.89 (4.16–5.31) mm	• Preoperative peripheral blood sample collection (plasma and serum);<br>• Gene (miR-4505, miR-4743-5p, miR-4750-3p) expression (RT-qPCR) analysis on plasma samples;	EAT: ↑ thickness Circulation: ↑ miR-4505, ↑ miR-4743-5p and ↓ miR-4750-3p. Altered miRNA expression used for risk stratification of Type 2 DM-CAD?

Diana Santos et al. (2026)<br><br>DOI: 10.3390/cells15020122	Cardiac surgery patients, n=64<br><br>CAD patients, n=26<br><br>DM, n=8<br>no-DM, n=18<br><br>No-CAD patients, n=38<br><br>DM, n=10<br>no-DM, n=28	CAD 26.5 ± 0.5<br><br>No-CAD 27.7 ± 0.6		• Intraoperative adipose tissue (EAT/SAT) collection;<br>• miRNA discovery expression (microarray) and pathway analysis on adipose tissue (EAT/SAT) samples (CAD-DM n=8, CAD-no-DM n=8, No-CAD-DM n=8, no-CAD-no-DM n=8);<br>• Validation of candidate miRNAs in EAT/SAT samples (RT-qPCR)	EAT vs SAT distinct miRNAs expression profile (dependent on DM status and not CAD presence). EAT: ↑ miR-155-5p as a pro-inflammatory signal (positive regulator of glucose uptake) and ↑ miR-324-5p (regulator of lipid accumulation and inflammation). SAT: ↑ miR-93a-3p (negative regulator brown adipocyte differentiation), ↑ miR-223-3p (adipocyte dysfunction and insulin resistance) and ↓ miR-324-5p (regulator of lipid accumulation and inflammation). Cardioprotecive tissue-specific effects of miR-324-5p?

Clinical studies - adipo-cytokines and immune mediators

Tomasz Mazurek et al. (2003)<br><br>DOI: 10.1161/01.CIR.0000099542.57313.C5	CABG patients, n=42	31 ± 1	N/A	• Preoperative peripheral blood sample collection (plasma and serum);<br>• Intraoperative adipose tissue (EAT/SAT) samples collection;<br>• Cultured adipose tissue (EAT/SAT) samples and conditioned media collection (3h);<br>• Immunohistochemistry on adipose tissue (EAT/SAT) samples (n=11);<br>• Protein (IL-6, IL-6sR, MCP-1, TNF-α, IL-1β, insulin) expression analysis (ELISA) on plasma/AT-conditioned media;<br>• Gene (MCP-1, IL1β, IL-6 and TNF-α) expression analysis (RT-qPCR, microarray, n=11) on adipose tissue (EAT/SAT) samples;	EAT: ↑ inflammatory mediators levels, ↓ adipocyte-related genes expression (vs SAT/plasma). EAT has a proinflammatory action independently of major risk factors (DM, BMI, drugs).
Adam R. Baker et al. (2006)<br><br>DOI: 10.1186/1475-2840-5-1	Cardiac surgery patients, n=103<br><br>CABG, n=46<br><br>No-CAD, n= 57	CABG 27.5 ± 3<br><br>No-CAD 25.7	N/A	• Preoperative peripheral blood sample collection (serum);<br>• Intraoperative EAT (CABG, n= 46)/ SAT (no-CAD, n=30), omental AT (no-CAD,	EAT: ↑ macrophage infiltration, similar adipokine expression profile vs OAT. EAT/circulation: ↑ CD45,

		± 5		n=14)/ gluteal AT (no-CAD, n=13) samples collection;<br>• Protein expression (insulin, adiponectin, leptin, CRP and resistin) analysis (ELISA) on serum samples;<br>• Gene expression (resistin, adiponectin, IL-6, CD45, AGT, TNF-α, PAI -1, t-PA and leptin) analysis (RT-qPCR) on AT (EAT/SAT/OAT/GAT) samples.	resistin and AGT, ↓ IL-6, adiponectin and PAI-1 in CABG vs no-CAD patients. ↓ IL-6 in CABG-statin treated patients vs CABG not treated patients. Pathogenic role of EAT and circulation inflammation in CAD onset and progression?
J. N. Fain et al. (2008)<br><br>DOI: 10.1038/sj.ijo.0803790	Cardiac surgery patients, n= 27<br><br>Bariatric surgery patients, n=13<br><br>Male patients, n=14<br><br>Female patients, n=13	Men 32 ± 2<br>Women 29 ± 1	N/A	• Intraoperative adipose tissue (EAT/SATs, n=27; OAT, n=13) samples collection;<br>• OAT-derived adipocytes/non fat-cells isolation (n=13);<br>• Gene expression (omentin, visfatin) analysis (RT-qPCR) on AT (EAT/SATs/OAT) samples and OAT-derived cells.	EAT: ↑ omenitin deposit-specific expression. Visfatin is uniformly present in all AT samples.

T. Rubicek et al. (2008)<br><br>DOI: 10.33549/physiol res.931315	Cardiac surgery patients, n= 10<br><br>CABG, n = 7<br><br>VR, n =3	27 ± 3	N/A	• Pre- and post-operative peripheral blood sample collection (serum);<br>• Pre- and post-operative adipose tissue (EAT/SAT) samples collection;<br>• Pre- and post-blood parameters (glucose, insulin and adiponectin) measurement (plasma/serum);<br>• Gene (AGT, ACE and AGTR1) expression analysis (RT-qPCR) on adipose tissue (pre-/post-EAT/SAT) samples	Circulation: ↑ glucose and insulin pre-operative vs post-operative<br>EAT: ↑ angiotensinogen expression during acute stress (surgery)<br>EAT expression of angiotensinogen leads to insulin resistance development?
Nicolas Venteclef et al. (2015)<br><br>DOI: 10.1093/eurheartj/eht 099	CABG patients, n = 39<br><br>Group 1, n=12 (transcriptomics)<br><br>Group 2, n=13 (organocolture)<br><br>Group 3, n=14 (proteomics)	G1 26.5 ± 3.1<br><br>G2 29.6 ± 6.6<br><br>G3 26.4 ± 4.4	N/A	• Intraoperative adipose tissue (EAT/SAT) and atrial (n=18) biopsies collection; Autoptic ventricular (n=5) biopsy collection;<br>• Cultured EAT/SAT samples and secretome collection;<br>• In vitro conditioning (EAT-/SAT-secretome) of rat atrial organo-culture (G2, n=13);<br>• Immunohistochemistry on rat atrial organo-culture samples (1 week) and atrial/ventricular human biopsies;<br>• Atrial fibroblast isolation (atrial biopsies) and ex vivo	EAT: ↑ angiogenetic factors, Activin A and MMP8 production and secretion (vs SAT) → ↑ fibrosis in adjacent myocardium

				conditioning (EAT-/SAT-secretome);<br>• Gene expression analysis (qRT-PCR) on EAT/SAT/organo-culture samples (G1, n = 12);<br>• Protein (adipo-cytokines) expression (ELISA/cytometric bead array) analysis on EAT/SAT secretome (G3, n=14)	
Vishal Vyas et al. (2021)<br><br>DOI: 10.1172/jci.insight.145495	Cardiac surgery patients, n=152<br><br>VR, n=54<br><br>CABG, n=82<br><br>CABG/VR, n=16	VR 27 ± 3<br><br>CABG 29 ± 3<br><br>CABG/VR 29 ± 2	N/A	• Preoperative peripheral blood sample collection;<br>• Intraoperative adipose tissue (EAT/SAT) collection;<br>• Immune cell profiling (flow cytometry) on adipose tissue (EAT/SAT) and blood samples;<br>• Immunohistochemistry on adipose tissue (EAT/SAT) and blood samples (CABG, n = 17 and VR, n = 8);<br>• Gene expression analysis (bulk RNA sequencing, qRT-PCR) on EAT and blood samples	EAT: ↑ IL-1, IL-6, TNF-α and IFN-γ expression in obesity and DM patients (regardless of CAD/hypertension status).

Olga V. Gruzdeva et al. (2022)<br><br>DOI: 10.3390/jpm12020129	Cardiac surgery patients, n=145<br><br>CABG patients, n=125<br><br>VR patients, n=120	CABG 28,9 ± 3<br><br>VR 26.8 ± 3	EAT thickness measurement (MRI)<br><br>CABG, n=125<br><br>LV (mm) 3.53 (2.87; 4.36)<br>RV (mm) 4.58 (4.09; 6.17)<br><br>VR, n=120<br><br>LV (mm) 2.76 (2.42; 3.21)<br>RV (mm) 3.65 (3.12; 3.97)	• Intraoperative adipose tissue EAT/SAT/PVAT) biopsies collection;<br>• AT-derived adipocytes isolation (EAT/SAT/PVAT samples) and culture medium collection (24h);<br>• Gene expression (adiponectin, leptin, leptin receptor and IL-6) analysis<br>• (RT-qPCR) on isolated adipocytes;<br>• Protein expression (adiponectin, leptin) analysis (ELISA) on culture medium.	EAT: ↑ leptin and IL-6 (pro-inflammation) expression and release and ↓ adiponectin (cardioprotective).<br>EAT altered expression profile → activation of immune cells → development and progression of atherosclerosis (CAD patients) ?
Serena Cabaro et al. (2022)<br><br>DOI: 10.3389/fcell.2022.893729	Cardiac surgery CAD patients, n=40<br><br>No-Post-operative AF (No-POAF), n=24<br><br>Post-operative AF (POAF), n=16	No-POAF 29 ± 2<br><br>POAF 29 ± 2	EAT thickness measurement (echocardiogram)<br><br>No-POAF, n=24<br>14,5 ± 2,3mm<br><br>POAF, n=16<br>14,5 ± 2,5 mm	• Preoperative blood sample collection (serum);<br>• Intraoperative EAT and atrial biopsy collection;<br>• Cultured EAT samples and conditioned media collection;<br>• Cytokine (IL-1β, IL- 1ra) assay on serum and EAT-conditioned media;<br>• In vitro conditioning (IL-1β/IL-18) of human atrial fibroblasts (atrial biopsy);<br>• Gene expression (IL-1β, Collagen 1A1, Collagen 3A1, TGF	EAT: ↑ IL-1β → ↑ Inflammatory stimuli (via NLRP3 inflammasome)→ pro-fibrotic and remodeling effect on fibroblast → ↑ IL-1β and TGF-β production<br>EAT-derived (not serum) IL-1β contribute in POAF onset?

				β1) analysis (qRT-PCR) on EAT and cell cultures	
Eva R. Meulendijks et al. (2023)<br><br>DOI: 10.1186/s12967-023-04231-2	AF patients, n=20<br><br>No-AF patients, n=35<br><br>Persistent AF, n=14<br><br>Paroxysmal AF, n=6<br><br>Future-onset AF, n=15	AF 29 ± 3.4<br><br>No-AF 27.5 ± 3.5	N/A	• Intraoperative EAT and atrial (LAA) biopsy collection;<br>• Cultured EAT samples and conditioned media collection (1h);<br>• In vitro conditioning (EAT-secretome, AF n=14, no-AF n=4) of NHCF-As;<br>• Gene expression (COL1A1, FN1) analysis (RT-qPCR) on conditioned NHCF-As (48h);<br>• Proteome analysis (LC–MS/MS) on EAT/EAT-secretome samples (AF n=3, no-AF n=3);<br>• Immunohistochemistry (MPO) on atrial (LAA) biopsies (P. AF n=10, Pa. AF n=6, F. AF n=15, No-F. AFr n=14)	EAT: ↑ MPO (profibrotic factor) secretion in AF patients → ↑ structural remodeling (ECM proteins: COL1A1 and FN1).<br>↑ MPO and neutrophils (NETs) in patients before AF onset.<br>Role in fibrotic substrate priming?

Evidence in human and animal model

Auriane C Ernault et al. (2022)<br><br>DOI: 10.1016/j.hrthm.2022.05.011	AF patients, n=30<br><br>Controls (no AF), n=3<br><br>Persistent AF, 97%<br><br>Long-standing persistent AF, 3%<br><br>DM 3% (67)<br>HF 10% (0)<br>HTN 47% (0)	AF 29 ± 4<br><br>No AF 30 ± 5	N/A	• Intraoperative adipose tissue (EAT/SAT) collection (AF patients);<br>• EAT collection from donated human hearts (controls no AF);<br>• Cultured EAT/SAT samples and secretome isolation (24h);<br>• In vitro conditioning (EAT/SAT/CCM secretome) of NRVMs (72h);<br>• Gene and protein expression analysis (qRT-PCR and western blot) on NRVMs;<br>• Electrophysiological experiments on NRVMs (patch-clamp at 48h, electrical mapping at 72h);<br>Arrhythmia inducibility test (in silico human LA model)	EAT secretome: arrhythmogenic effect on cardiac tissue (via depolarization of resting membrane, conduction slowing and increased conduction heterogeneity).<br>*Kcnj2/connexin43* as molecular targets of EAT secretome →arrhythmogenic effect?
Olga Shaihov-Teper et al. (2021)<br><br>DOI: 10.1161/CIRCULATIONAHA.120.052009	Elective cardiac surgery patients, n=62<br><br>AF patients, n=32<br><br>Control no AF, n=30	AF 26.1 ± 3.3<br><br>No-AF 28.1 ± 3.9	N/A	• Intraoperative EAT sample collection;<br>• Cultured EAT samples and conditioned media collection (at 2-4-6-9 days);<br>• Immunohistochemistry on adipose tissue (fresh EAT/cultured EAT) samples;<br>• EAT-EVs isolation (ultracentrifuge), purification (size exclusion chromatography, sucrose cushion) from EAT-cultured media.<br>• EAT-EVs	EAT-EVs: highly secreted and enriched in cytokines, proteins, miR-146b, miR-133a and mir-29a in AF patients.<br>EV-cargo→ proinflammatory, profibrotic and proarrhythmic effect → sustained AF.<br>Causal role of EAT-EVs in AF onset by

				characterization: concentration and dimension (flow cytometry, NTA), morphology (TEM), specific markers (western blot), encapsulated cytokine (cytokine array, ELISA), proteome (mass spectrometry), miRNA content (RT-qPCR)<br>• In vitro conditioning (EAT-EVs) of atrial MSCs, HUVEC and iPSC-CM cultures<br>• In vivo injection (EAT-EVs) in rat hearts	mediating cellular and electric remodeling of the myocardium?
Auriane C Ernault et al. (2023)<br><br>DOI: 10.1016/j.hroo.2023.10.007	AF patients, n=17	28 ± 3	N/A	• Intraoperative adipose tissue (EAT/SAT) collection (n=17);<br>• Cultured EAT/SAT samples and secretome isolation;<br>• EVs quantification (flow cytometry, n=7)<br>• EAT-EVs isolation (size exclusion chromatography, n=8);<br>• miRNAs sequencing and pathway analysis (n=8);<br>• Validation of candidate miRNAs in EAT/SAT/NRMV samples (RT-qPCR, n=8);<br>• Overexpression of candidate miRNAs in NRVMs (miRNA mimics transfection);	EAT-EVs: miR-1-3p and miR-133a-3p<br>Rat model: ↑ miR-1-3p and miR-133a-3p →↓ conduction and ↓ *Kcnj2*/*Kcjn12* expression (potassium channels).<br>miR-1-3p and miR-133a-3p in EAT-EVs as mediators of arrhythmogenic effect on cardiac tissue (via depolarization of resting membrane, conduction slowing and

				• Electrophysiological experiments on NMRVs (at 48h)	increased conduction heterogeneity)?
Maria Cristina Carena et al. (2023)<br><br>DOI: 10.1016/j.jacc.2023.05.031	Cardiac surgery patients, n=1246<br><br>Study arm 1, n= 6<br><br>Study arm 2, n= 429<br><br>Study arm 3, n= 344<br><br>Study arm 4, n= 5<br><br>Study arm 5, n= 462	SA1 29.8 ± 1.9<br><br>SA2 28.4 ± 0.2<br><br>SA3 28.2 ± 0.2<br><br>SA4 28.1 ± 2.0<br><br>SA5 28.3 ± 0.2	N/A	• Peripheral whole blood collection (S3);<br>• Intraoperative EAT (SA1+SA2) and atrial (RAA, SA1+SA2+SA4) biopsies collection;<br>• Cultured (WNT5A/ SFRP5) atrial (RAA) biopsies;<br>• Cultured EAT samples and secretome collection;<br>• miRNA profiling (microarray) and pathway analysis on EAT/EAT-secretome samples (SA1);<br>• Validation of candidate miRNAs in EAT samples (RT-qPCR, S2 n=206);<br>• In vitro expression (miRNA mimics transfection) of candidate miRNAs (miR-30c-5p, miR-92a-3p, miR-193a-5p) in H9c2/RAAs and murine heart samples;<br>• Genome-wide genetic screening on blood samples (SA3);<br>• In vitro expression (miRNA	EAT: Production and secretion of miR-92a-3p (cardioprotective).<br>↓ redox status via miR-92a-3p/ WNT5A/Rac1/ NADPH oxidase.<br>↑ miR-92a-3p→↓ risk of POAF onset

				mimics transfection) of candidate miRNAs (miR-30c-5p, miR-92a-3p, miR-193a-5p) in H9c2/RAAs and murine heart samples;<br>• In vivo (mouse) expression of Wnt5a experiment and murine heart collection;<br>• Superoxide production measurements (chemiluminescence) on cell cultures, RAA and murine hearts samples;<br>• Gene expression analysis (RT-qPCR)<br>• Protein expression analysis (western blot) of cell cultures;	
